# Phase estimation algorithm for the multibeam optical metrology

**DOI:** 10.1038/s41598-020-65466-3

**Published:** 2020-05-26

**Authors:** V. V. Zemlyanov, N. S. Kirsanov, M. R. Perelshtein, D. I. Lykov, O. V. Misochko, M. V. Lebedev, V. M. Vinokur, G. B. Lesovik

**Affiliations:** 1 Moscow Institute of Physics and Technology, 141700 Institutskii Per. 9, Dolgoprudny, Moscow Distr. Russian Federation; 2 Consortium for Advanced Science and Engineering (CASE), University of Chicago, 5801 S Ellis Ave, Chicago, IL 60637 USA; 3 Low Temperature Laboratory, Department of Applied Physics, Aalto University, P.O. Box 15100, FI-00076 Aalto, Finland; 4 Institute of Solid State Physics, Russian Academy of Sciences, 142432 Chernogolovka, Moscow Distr. Russian Federation; 5 Materials Science Division, Argonne National Laboratory, 9700 S. Cass Ave., Argonne, IL 60439 USA

**Keywords:** Optoelectronic devices and components, Information theory and computation

## Abstract

Unitary Fourier transform lies at the core of the multitudinous computational and metrological algorithms. Here we show experimentally how the unitary Fourier transform-based phase estimation protocol, used namely in quantum metrology, can be translated into the classical linear optical framework. The developed setup made of beam splitters, mirrors and phase shifters demonstrates how the classical coherence, similarly to the quantum coherence, poses a resource for obtaining information about the measurable physical quantities. Our study opens route to the reliable implementation of the small-scale unitary algorithms on path-encoded qudits, thus establishing an easily accessible platform for unitary computation.

## Introduction

Unitary Fourier transform is a quintessential component for a multitude of quantum computational algorithms^1–3^ as it underlies a versatile phase estimation routine^4^ which is at the core of various quantum metrological protocols^5–8^. Such phase-sensitive protocols, utilizing coherence for measurements of physical quantities, find use in quantum sensors^9,10^, notably in the qudit-based devices (e.g., based on the superconducting artificial atoms or NV centers) for determining magnetic and electric fields^11–17^. Importantly, since these protocols do not necessarily employ quantum entanglement^18^, they may be implemented on the systems that manifest wave yet classical behavior. Therefore, methods borrowed from quantum metrology can be applied to the classical optical phase measurements^19–23^, which, in particular, can be used to measure the position, velocity, and displacement of physical objects. Here we report on constructing a complex linear-optic-based device capable to carry out the Fourier-based phase estimation algorithm. The metrological potential of the intricate multiple-beam interference schemes can be, for instance, seen in the LIGO optical gravitational wave detector where the Heisenberg-limited sensitivity is achieved through combining Michelson and Fabry-Pérot interferometers and employing the squeezed states of light.

Our approach is predicated upon the fact that any finite-dimensional unitary matrix can be realized by means of 50:50 beam splitters, phase shifters and mirrors^24^. In order to better demonstrate the computational capabilities of the linear optics, we adopt the laser as a source of the light having the coherence length by far exceeding the size of the setup. This ensures the speed of measurements that is sufficient to support the stability of the interference pattern during the time necessary for collecting the required statistics. Note that in the single-photon regime, the time needed to obtain the same statistics would be much too long to preserve the same quality of the interference pattern throughout the entire measurement procedure. Using the multiphoton source does not eliminate the unitary nature of the algorithm, which employs for this moderate computation scale only the wave aspect of the signal. Switching to the single-photon source for practical computation purposes will translate the scheme into the fully quantum one, while maintaining the major characteristics manifested by the present device. A general architecture for such a multiport interferometer was first proposed by Reck *et al*.^24^ and then further reframed by Clements *et al*.^25^. The theoretical prospects of the proposed architecture were discussed in refs. ^26,27^. Experimentally, it was shown that linear optical protocols can be implemented on a photonic chip^22,28^. Yet, the practical engineering of such a structure remains highly challenging.

In what follows, we will overview our algorithm and the theoretical background, describe our experimental layout, and construct the analytical description of the computational scheme. Finally, we discuss the results and outline the future research directions.

## Preliminaries

### Algorithm description

We start with the description of the Fourier phase-estimation algorithm operating in the qudit regime. The initial qudit state is taken as a superposition of all computational states:

1 $$|{\Psi }_{\phi }\rangle =\frac{1}{\sqrt{d}}\,\mathop{\sum }\limits_{k=0}^{d-1}\,{e}^{ik\phi }|k\rangle ,$$

where $\{|k\rangle {\}}_{k=0}^{d-1}$ is an orthonormal computational basis in the qudit’s Hilbert space. Additionally, we let $\phi =\frac{2m\pi }{d}$, $m\in \{0,1,\ldots ,d-1\}$. The algorithm has to unambiguously determine the value of $\phi$ via a single-shot measurement of the qudit state. This is achieved by applying a base-*d* quantum Fourier transformation with the corresponding unitary operator $\hat{F}$,

2 $$\hat{F}|n\rangle =\frac{1}{\sqrt{d}}\,\mathop{\sum }\limits_{k=0}^{d-1}\,{e}^{-2\pi ink/d}|k\rangle .$$

The action of $\hat{F}$ on the initial state $|{\Psi }_{\phi }\rangle$ yields one of the states from the computational set $\{|k\rangle {\}}_{k=0}^{d-1}$ depending on $\phi =\frac{2\pi m}{d}$:

3 $$|{\Psi }_{{\rm{out}}}\rangle =\hat{F}|{\Psi }_{\phi }\rangle =|m\rangle .$$

Accordingly, by measuring the output state $|{\Psi }_{{\rm{out}}}\rangle$ one determines the value of $\phi$.

The above algorithm appears as a subroutine in a family of conditional sequential sensing protocols with the scaling corresponding to the Heisenberg limit, for example, the Kitaev protocol. An essential principle of these protocols is the phase encoding: on each step of the procedure, the state of the qudit is tagged with the phase $\phi$ (as in Eq. (1)) which depends on the unknown constant physical value to be determined and on the sensing period of the step *t*.

### Optical scheme

Now we introduce our optical framework. In this setting the qudit is represented by the *d* coherent beams. Each element of its *d*-dimensional state vector is a complex amplitude of the corresponding beam. Accordingly, the state vector transforms when the light passes through the arrangement of beam splitters, phase shifters and mirrors. The task of constructing a particular unitary operator reduces to its decomposition into a sequence of the two-dimensional beam splitter transformations and individual phase shifts. In this section we devise base-3 (qutrit) scheme to carry out the Fourier transformation

4 $$\hat{F}=\frac{1}{\sqrt{3}}(\begin{array}{ccc}1 & 1 & 1\\ 1 & {e}^{4\pi i/3} & {e}^{2\pi i/3}\\ 1 & {e}^{2\pi i/3} & {e}^{4\pi i/3}\end{array}).$$

The matrix ${\hat{A}}_{jk}^{\chi }(\alpha ,\theta )$ of an arbitrary lossless beam splitter with the *j*th and *k*th input beams is expressed in the form

5 $${\hat{A}}_{01}^{\chi }(\alpha ,\theta )=(\begin{array}{ccc}\cos \chi \,{e}^{i\theta } & \sin \chi \,{e}^{i(\theta +\alpha )} & 0\\ -\sin \chi \,{e}^{i(\theta -\alpha )} & \cos \chi \,{e}^{i\theta } & 0\\ 0 & 0 & 1\end{array});$$

6 $${\hat{A}}_{12}^{\chi }(\alpha ,\theta )=(\begin{array}{ccc}1 & 0 & 0\\ 0 & \cos \,\chi \,{e}^{i\theta } & \sin \,\chi \,{e}^{i(\theta +\alpha )}\\ 0 & -\sin \,\chi \,{e}^{i(\theta -\alpha )} & \cos \,\chi \,{e}^{i\theta }\end{array}),$$

where $\chi$ determines the split ratio ($\sqrt{T}=\,\cos \,\chi$, $\sqrt{R}=\,\sin \,\chi$); *α* and *θ* are certain phases. The matrix ${\hat{P}}_{j}^{\beta }$ corresponding to the phase change by *β* of the *j*-th beam is defined as

7 $${\hat{P}}_{0,1,2}^{\beta }=\{(\begin{array}{ccc}{e}^{i\beta } & 0 & 0\\ 0 & 1 & 0\\ 0 & 0 & 1\end{array});\,(\begin{array}{ccc}1 & 0 & 0\\ 0 & {e}^{i\beta } & 0\\ 0 & 0 & 1\end{array});\,(\begin{array}{ccc}1 & 0 & 0\\ 0 & 1 & 0\\ 0 & 0 & {e}^{i\beta }\end{array})\}.$$

In order to prepare a beam splitter matrix with an arbitrary desired ratio of reflection to transmission, one has to assemble a Mach–Zehnder interferometer using two symmetric 50:50 beam splitters (for convenience, hereinafter we will omit the notation for dependence on $\alpha$ and $\theta$ if $(\alpha ,\theta )=(\pi /2,0)$):

8 $${\hat{A}}_{01}^{\chi }\equiv {\hat{A}}_{01}^{\chi }(\pi /2,0)={\hat{P}}_{0}^{\chi +\pi }\,{\hat{P}}_{1}^{\chi +3\pi /2}\,{\hat{A}}_{01}^{\pi /4}\,{\hat{P}}_{0}^{\pi -2\chi }\,{\hat{A}}_{01}^{\pi /4}\,{\hat{P}}_{1}^{\pi /2},$$

with ${\hat{A}}_{01}^{\pi /4}$ corresponding to the ideal symmetric beam splitter.

**Figure 1:**
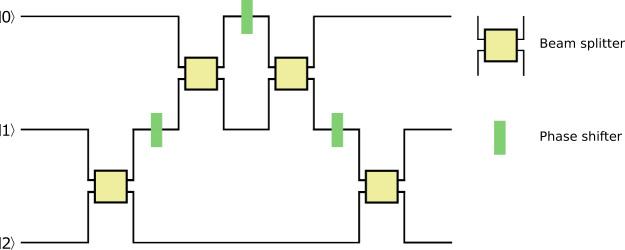
Optical circuit realizing the qutrit quantum Fourier transformation.

As shown in Ref. ^7^, the Fourier transformation $\hat{F}$ can be factorized as follows:

9 $$\begin{array}{rcl}\hat{F} & = & {\hat{P}}_{1}^{\pi /2}\,{\hat{A}}_{12}^{\pi /4}\,{\hat{P}}_{0}^{\pi }\,{\hat{A}}_{01}^{\tilde{\chi }}\,{\hat{P}}_{0}^{\pi }\,{\hat{P}}_{1}^{\pi /2}\,{\hat{A}}_{12}^{\pi /4}\,{\hat{P}}_{2}^{3\pi /2}\\  & = & {\hat{P}}_{0}^{\tilde{\chi }+\pi }\,{\hat{P}}_{1}^{\pi /2}\,{\hat{A}}_{12}^{\pi /4}\,{\hat{P}}_{1}^{\pi /2+\tilde{\chi }}\,{\hat{A}}_{01}^{\pi /4}\,{\hat{P}}_{0}^{\pi -2\tilde{\chi }}\,{\hat{A}}_{01}^{\pi /4}\,{\hat{A}}_{12}^{\pi /4}\,{\hat{P}}_{2}^{3\pi /2};\end{array}$$

where $\tilde{\chi }={\tan }^{-1}(\sqrt{2})$. It is seen from this expression that the experimental realization of $\hat{F}$ requires no more than 4 symmetric 50:50 beam splitters. The optical circuit for $\hat{F}$ is depicted in Fig. 1.

### Experimental setup

**Figure 2:**
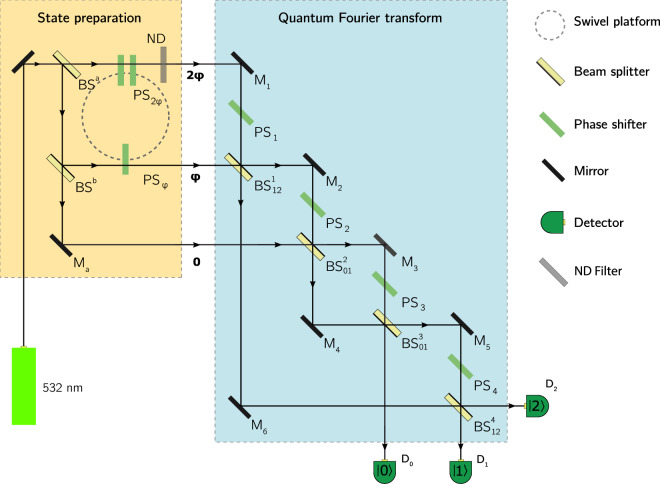
Experimental scheme for the qutrit case of the metrological algorithm.

The experimental layout is divided into two modules, as shown in Fig. 2. In the state preparation module, the incident laser beam is converted into the qutrit initial state given by Eq. (1). The beam splitters BS^*a*^ and BS^*b*^ generate three beams each representing a particular basis state $|j\rangle$ ($j=\{0,1,2\}$). The $|1\rangle$ and $|2\rangle$ beams then pass through respectively one (${{\rm{PS}}}_{\phi }$) and two (${{\rm{PS}}}_{2\phi }$) phase shifters attached to a swivel platform which sets the relative phases 0, $\phi$ and $2\phi$. The value of $\phi$ depends on the position of the platform: by rotating the platform one alters the length of the optical paths through the phase shifters and, therefore, changes $\phi$ without affecting the ratio between the relative phases.

The primary module shown in Fig. 2 realizes Eq. (9). However, although Eq. (9) directly translates the Fourier transformation into the optical setting, it fails to take account of limitations intrinsic to the real equipment. Namely, the transmission the phase shifters is associated with the intensity losses. In order to take such losses into account we should employ the corresponding operators ${\hat{L}}_{0,1,2}^{t}$:

$${\hat{L}}_{0,1,2}^{t}=\{(\begin{array}{ccc}t & 0 & 0\\ 0 & 1 & 0\\ 0 & 0 & 1\end{array});\,(\begin{array}{ccc}1 & 0 & 0\\ 0 & t & 0\\ 0 & 0 & 1\end{array});\,(\begin{array}{ccc}1 & 0 & 0\\ 0 & 1 & 0\\ 0 & 0 & t\end{array})\},$$

where $t$ is the absolute value of the transmission coefficient of and individual phase shifter. After the appropriate alignments, the equation for the operation realized in the primary module assumes the form

10 $$\begin{array}{ccc}\hat{U} & = & {[{\hat{A}}_{12}^{{\chi }_{0}}({\alpha }_{4},{\theta }_{4}){\hat{P}}_{2}^{{\psi }_{6}}{\hat{P}}_{1}^{{x}_{4}}{\hat{L}}_{1}^{{t}_{{\rm{p}}{\rm{s}}}}{\hat{L}}_{2}^{{t}_{{\rm{p}}{\rm{s}}}}{\hat{P}}_{1}^{{\psi }_{5}}]}_{4}\\  &  & \times \,{[{\hat{A}}_{01}^{{\chi }_{0}}({\alpha }_{3},{\theta }_{3}){\hat{P}}_{1}^{{\psi }_{4}}{\hat{P}}_{0}^{{x}_{3}}{\hat{L}}_{0}^{{t}_{{\rm{p}}{\rm{s}}}}{\hat{L}}_{1}^{{t}_{{\rm{p}}{\rm{s}}}}{\hat{P}}_{0}^{{\psi }_{3}}]}_{3}\\  &  & \times \,{[{\hat{A}}_{01}^{{\chi }_{0}}({\alpha }_{2},{\theta }_{2}){\hat{P}}_{1}^{{x}_{2}}{\hat{L}}_{1}^{{t}_{{\rm{p}}{\rm{s}}}}{\hat{P}}_{1}^{{\psi }_{2}}]}_{2}\\  &  & \times \,{[{\hat{A}}_{12}^{{\chi }_{0}}({\alpha }_{1},{\theta }_{1}){\hat{L}}_{2}^{{t}_{{\rm{p}}{\rm{s}}}}{\hat{P}}_{2}^{{x}_{1}}{\hat{P}}_{2}^{{\psi }_{1}}]}_{1},\end{array}$$

where ${t}_{{\rm{ps}}}$ is the modulus of the transmission coefficient of PS_1_, …, PS_4_; ${\chi }_{0}$ defines the beam splitters’ split ratio ($\sqrt{T}=\,\cos \,{\chi }_{0}$, $\sqrt{R}=\,\sin \,{\chi }_{0}$); ${\alpha }_{i}$ and ${\theta }_{i}$ correspond to BS^*i*^ (see Eq. (5)); ${\psi }_{i}$ is the phase change due to reflection of M_*i*_; ${x}_{i}$ is the phase change on PS_*i*_. In our experiment ${t}_{{\rm{ps}}}=0.935$, $T=0.445$ and $R=0.555$. The notation ${[\ldots ]}_{i}$ will be used later. For simplicity, the above formula does not explicitly include discrepancies in the optical distances. In this respect, we should define ${x}_{i}$ as a relative phase in which such terms along with the phase shift on PS_*i*_ are taken into account. The output state vector can be written as

11 $$|{\Psi }_{{\rm{out}}}({x}_{1},{x}_{2},{x}_{3},{x}_{4},\phi )\rangle =\hat{U}\{{\hat{P}}_{0}^{{\psi }_{a}}{\hat{L}}_{1}^{{t}_{\phi }}{\hat{P}}_{1}^{\phi }{\hat{L}}_{2}^{{t}_{ND}}{\hat{L}}_{2}^{{t}_{2\phi }}{\hat{P}}_{2}^{2\phi }{\hat{A}}_{01}^{{\chi }_{0}}({\alpha }_{a},{\theta }_{a}){\hat{A}}_{02}^{{\chi }_{0}}({\alpha }_{b},{\theta }_{b})|2\rangle {\}}_{{\rm{sp}}},$$

where the brackets ${\{\ldots \}}_{{\rm{sp}}}$ denote the state prepared in the first module of the scheme; ${t}_{\phi }$ are ${t}_{2\phi }$ are the absolute values of the transmission coefficient of ${{\rm{PS}}}_{\phi }$ and ${{\rm{PS}}}_{2\phi }$ respectively (in our experiment ${t}_{\phi }=0.875$, ${t}_{2\phi }=0.894$); ${t}_{ND}=0.837$ is the modulus of the transmission coefficient of neutral-density (ND) filter, used for leveling of the intensities; $({\alpha }_{a},{\theta }_{a})$, $({\alpha }_{b},{\theta }_{b})$ and ${\psi }_{a}$ correspond respectively to BS^*a*^, BS^*b*^ and M_*a*_.

For certain values of ${x}_{i}$ which we denote by ${x}_{i}^{F}$ and which are given by

12 $$\begin{array}{rcl}{x}_{1}^{F} & = & -{\alpha }_{1}+{\alpha }_{a}-{\alpha }_{b}+{\theta }_{b}-{\psi }_{1}+\pi ;\\ {x}_{2}^{F} & = & -{\alpha }_{2}+{\alpha }_{b}-{\theta }_{1}+{\psi }_{a}-{\psi }_{2}-\pi /2;\\ {x}_{3}^{F} & = & -{\alpha }_{2}+{\alpha }_{3}-{\psi }_{3}+{\psi }_{4}+\pi -2\tilde{\chi };\\ {x}_{4}^{F} & = & -{\alpha }_{1}+{\alpha }_{2}+{\alpha }_{4}-{\alpha }_{b}+{\theta }_{1}-{\theta }_{2}-{\theta }_{3}-{\psi }_{4}-{\psi }_{5}+{\psi }_{6}-{\psi }_{a}-\pi +\tilde{\chi },\end{array}$$

the transformation implemented in the scheme is similar to Eq. (9):

13 $${\hat{F}}_{exp}={[{\hat{A}}_{12}^{{\chi }_{0}}{\hat{P}}_{1}^{\pi \mathrm{/2}+\tilde{\chi }}{\hat{L}}_{1}^{{t}_{{\rm{ps}}}}{\hat{L}}_{2}^{{t}_{{\rm{ps}}}}]}_{4}\,{[{\hat{A}}_{01}^{{\chi }_{0}}{\hat{P}}_{0}^{\pi -2\tilde{\chi }}{\hat{L}}_{0}^{{t}_{{\rm{ps}}}}{\hat{L}}_{1}^{{t}_{{\rm{ps}}}}]}_{3}\,{[{\hat{A}}_{01}^{{\chi }_{0}}{\hat{L}}_{1}^{{t}_{{\rm{ps}}}}]}_{2}\,{[{\hat{A}}_{12}^{{\chi }_{0}}{\hat{L}}_{2}^{{t}_{{\rm{ps}}}}{\hat{P}}_{2}^{3\pi \mathrm{/2}}]}_{1}\mathrm{}.$$

**Figure 3:**
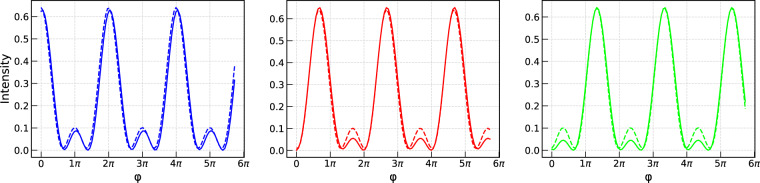
Theoretical plots of the intensity on the $|0\rangle$ (blue line), $|1\rangle$ (red line) and $|2\rangle$ (green line) detectors as functions of $\phi$. Dashed line shows the results obtained by means of Eq. (9), whereas the solid line refers to Eq. (13).

Here we ignored the phases of the resulting beams incident on the detectors. For the description of the alignment procedure see Methods and SI. Figure 3 displays the theoretical plots obtained using Eq. (9) (dashed lines) and (13) (solid lines). Both series of plots are almost identical. Note, that taking losses into account in Eq. (13) results in smaller secondary peaks.

## Results and Discussion

**Figure 4:**
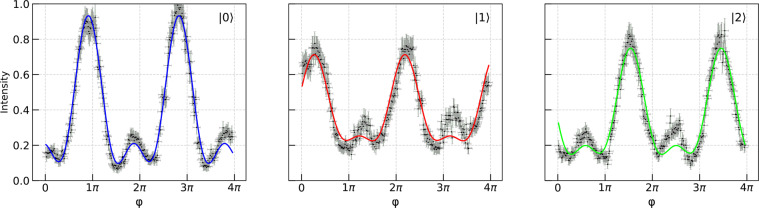
The measured intensities on each of the detectors as functions of $\phi$. The solid line shows the theoretical fit to the data. Each data point is calculated by averaging the experimental signal on the detector over ~0.5 s with the fixed angle of the swivel platform; the vertical error bars represent the corresponding signal dispersion. The horizontal error bars reflect the precision limit of the swivel platform.

Figure 4 shows the measured intensities as functions of $\phi$. The data on D_*i*_ are fit by the square of *i*th element of the output vector function given by Eq. (11):

14 $${p}_{i}={a}_{i}{|\langle i|{\Psi }_{{\rm{o}}{\rm{u}}{\rm{t}}}({x}_{1},{x}_{2},{x}_{3},{x}_{4},\kappa \cdot \phi +\mu )\rangle |}^{2}+{b}_{i},$$

where ${a}_{i}$ is the intensity scaling parameter; ${b}_{i}$ is the intensity bias simulating the interference visibility loss; $\kappa$ and $\mu$ are respectively the phase scaling parameter and phase shift independent of $i$. The fitting is done using the method of least squares. Note, that the same unitary transformation can be realized with the different sets of parameters. For details on fitting and determining the corresponding errors see SI and Methods. The phases ${x}_{i}$ determined from the fit are given by

15 $$({x}_{1},{x}_{2},{x}_{3},{x}_{4})=({x}_{1}^{F},{x}_{2}^{F},{x}_{3}^{F},{x}_{4}^{F})\pm (0.28,0.30,0.30,0.28),$$

where the second term is the error of fitting. Despite the discrepancies (which, as a matter of fact, are small as compared to *π*) described by the second term, our data compare fairly well with the theoretical plots presented in Fig. 3. The results show that the interference is controlled to the high degree in spite of the complexity of the optical scheme. Thus, the described optical platform proves to be capable to perform small scale unitary operations.

Note that our experiment is carried out in a multiphoton rather than in a single-photon regime which typically serves as a bedrock for the optical implementations of the quantum algorithms. At the same time, similarly to many existing quantum algorithms, the realized Fourier phase-estimation protocol relies on the wave interference effects although it does not utilize specifically quantum phenomena. As any lossless quantum computation, the Fourier transform is described by the unitary operator^4^. We have constructed such a unitary operator through the specific arrangement of linear optical elements. It should be noted, however, that the discussed multiphoton approach does not support the algorithms relying on choosing between quantum alternatives (which takes place, for example, in the quantum random number generation procedure).

As shown in ref. ^24^, the number of beam splitters needed to construct a general $N$-dimensional unitary matrix $U$ grows as $N(N-1)/2$. The practical realization of such a multiport architecture, however, imposes additional scalability limitations (see SI for the detailed quantitative analysis):

1. *Restricted phase adjustment precision*. The relative cumulative error in the constructed matrix $U$ caused by the limited precision Δ*α* with which we control the rotation angle of the optical holders and the width $d$ of the phase shifters is of order *N(d/λ)(n-1)*Δ*α*. Here *n* is the refractive index of the phase shifters, and* λ* is the light wavelength.
2. *Restricted precision of the wavefronts*’ *alignment*. The misalignment of the wavefronts results in the complex interference pictures which can no longer be considered one-dimensional. The visibility of the picture deteriorates with the factor ≈$1-\frac{N{\left(R\Delta \alpha \frac{2\pi }{\lambda }\right)}^{2}}{8}$, where $R$ is the size of the beam spot.
3. *Phase fluctuations caused by the surface roughness*. Assuming that the light acquires the delta-correlated random phase $\delta \xi$ due to the surface roughness of the optical elements, we estimate the corresponding visibility deterioration factor as ~${e}^{-N\langle \delta {\xi }^{2}\rangle }$.
4. *Intensity losses*. The intensity losses on the mirrors and beam splitters used in our experiment are about 1%, which is acceptable. By far larger losses (≈10%) are associated with the phase shifters. Nevertheless, the use of anti-reflective coating would reduce these losses to 1%. The signal intensity on the detector would be ${0.99}^{N}\approx \exp (\,-\,N/100)$.

Based on these estimates, a detailed quantitative analysis devises the prospect for realizing matrices with $N$ up to of order 100 (this upper limit is set mostly by item 4, for other details see SI). This improvement will be built on the enhanced experimental and theoretical framework comprising the advanced adjustment precision of optical holders, eliminating the elements’ surface roughness, minimizing intensity losses (e.g., via employing the anti-reflective coating), and mitigating the drift of phases caused by the mechanical oscillations and instability of the optical elements. Note that the latter issue results in the rising deviation between the data and the fit as seen in Fig. 3. The corresponding improvement will be achieved by implementing the mechanical feedback phase control.

Further refining the concert between the theoretical description and the experimental realization will be achieved via including into the scheme the machine learning algorithms capable to compensate the imprecision in the alignment of the optical elements. These techniques have already passed the reliability test in the base-4 (ququart) version of the setup which we have already successfully realized. The obtained results manifest the improved accuracy and serve as the evidence of the scheme’s scalability. The detailed description of the ququart experiment will be the subject of the forthcoming publication.

There have been a recent progress in demonstrating the advantage of Quantum Fourier transforms (QFT) interferometers using both path and polarization modes^29^ and in realizing interferometric phase estimation algorithm approaching the Heisenberg limit^30^. Our scheme employs larger number of linear optic elements as compared to above references and utilizing path modes only. Yet we achieved a fairly high level of the correspondence between the experiment and theory. By adding the polarization degrees of freedom analogously to^29,30^, we will further increase the dimension of the unitary matrix realized by our scheme.

## Methods

Our optical setup includes the following equipment:

### Phase shifters

The phase shifters mainly serve to adjust the relative phases of the beams. In our setup, we use pieces of thick glass; the intensity loss on these elements is near $\mathrm{12.5 \% }$.

### Beam splitters

We employ beam splitters with dielectric coating optimized for the 400–700 nm range. The nominal split ratio is 50:50. In practice however, this holds only if the incident laser beam is unpolarized. For the case of the linearly polarized beam used in our experiment, the split ratio is close to 55:45.

### Mirrors

Dielectric mirrors optimized for the 400–700 nm range.

### Laser

Diode pumped solid state laser, 532 nm, 150 mW; the coherence length of light is 50 m.

### Detectors

Photodiode detectors.

The use of the photodiodes for the detection is justified by their high measurement speed (as opposed to the single-photon detectors), as the main goal of the present work was to test the interference capacity of the complex optical setup. However, an additional testing series employing the single-photon detectors has shown practically the same results as presented. This fact means that such a replacement does not pose any significant changes in the operation of the circuit.

**Figure 5:**
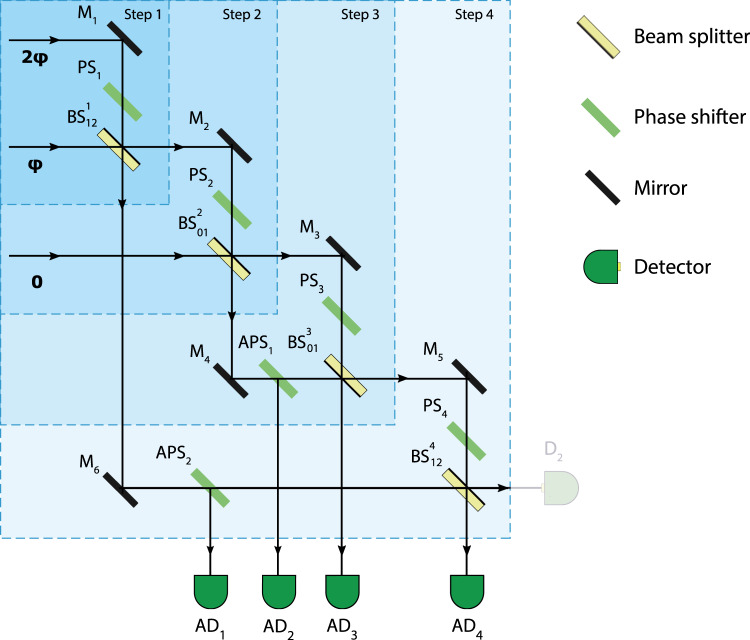
Four consecutive steps of the alignment procedure. At *i*th step, the output signal from the corresponding sector of the scheme (measured by the detector $A{D}_{i}$) is tuned to comply with the theoretical value calculated through the breakdown of Eq. (13). The tuning is done through the alignment of $P{S}_{i}$.

The alignment of the scheme is done in accordance with a step-by-step procedure which lays in tuning the signal at the intermediate points of the beams’ paths (see Fig. 5). At each consecutive step, the interference intensity at the given point is matched with the theoretical value obtained through the breakdown of Eq. (13): the *i*th step of the procedure leverages the *i*th block of operators (${[\ldots ]}_{i}$) in the relation. At the first two stages, we receive the signal reflected from the phase shifters PS_2_ and PS_3_ using the detectors AD_1_ and AD_2_, respectively. In turn, the last two stages involve the signals from the detectors AD_3_ and AD_4_. The alignment is performed via rotating the phase shifters (i.e., altering the optical path length) preceding the given point. By doing so, one changes the phases ${x}_{i}$ which in the end should be equal to ${x}_{i}^{F}$ given by Eq. (12). For details see SI.

Each point in Fig. 4 is obtained by averaging signals from the detectors generated over ~0.5 s. The oscillations and instability of the optical elements are represented by the vertical error bars. To estimate the corresponding error, we measured the signal discrepancies appearing over a characteristic period of time (~0.5 s) with the fixed angle of the swivel platform (determining the value of $\phi$). The horizontal error bars express the limited precision of the swivel platform.

The fitting of the experimental data is done via applying the method of least squares, see more detail in SI. The phases ${\bf{x}}={[{x}_{1},{x}_{2},{x}_{3},{x}_{4}]}^{T}$ corresponding to the optimal fit turned out to be very close to ${{\bf{x}}}^{F}={[{x}_{1}^{F},{x}_{2}^{F},{x}_{3}^{F},{x}_{4}^{F}]}^{T}$. The fitting error of ${x}_{k}$ ($k\in \{1,2,3,4\}$) is determined by the maximum size of the neighbourhood ${{\mathcal{O}}}_{k}^{F}$ of ${x}_{k}^{F}$ such that for any ${\tilde{x}}_{k}\in {{\mathcal{O}}}_{k}^{F}$ the standard deviation of ${p}_{i}(\tilde{{\bf{x}}},\phi )$ (with $\tilde{{\bf{x}}}={[{x}_{1}^{F},\ldots ,{\tilde{x}}_{k}^{F},\ldots ,{x}_{4}^{F}]}^{T}$) from ${p}_{i}({{\bf{x}}}^{F},\phi )$ ($i\in \{0,1,2\}$) does not exceed the experimental error.

## Supplementary information

Supplementary Information.

## Data Availability

All data generated or analyzed during this study are included in this published article and its Supplementary Information file.
