# Supplementary material for: Phase estimation algorithm for the multibeam optical metrology
Source: Sci Rep. 2020 May 26;10:8715. doi: 10.1038/s41598-020-65466-3 (PMC7251105; doi:10.1038/s41598-020-65466-3)
Supplement: Supplementary file 1 — Supplementary Information. [file 41598_2020_65466_MOESM1_ESM.pdf]

# Supplementary Information: Phase estimation algorithm for the multibeam optical metrology

V. V. Zemlyanov[1], N. S. Kirsanov[1,2,3], M. R. Perelshtein[1,3], D. I. Lykov[1], O. V. Misochko[4,1], M. V. Lebedev[4,1], V. M. Vinokur[5,2,*], and G. B. Lesovik[1]

[1]Moscow Institute of Physics and Technology, 141700, Institutskii Per. 9, Dolgoprudny, Moscow Distr., Russian Federation
[2]Consortium for Advanced Science and Engineering (CASE), University of Chicago, 5801 S Ellis Ave, Chicago, IL 60637, USA
[3]Low Temperature Laboratory, Department of Applied Physics, Aalto University, P.O. Box 15100, FI-00076 AALTO, Finland
[4]Institute of Solid State Physics, Russian Academy of Sciences, 142432, Chernogolovka, Moscow Distr., Russian Federation
[5]Materials Science Division, Argonne National Laboratory, 9700 S. Cass Ave., Argonne, IL 60439, USA
[*]vinokour@anl.gov

May 4, 2020

## Alignment

In this section we describe the alignment procedure for the qutrit quantum Fourier transformation setup. Our step-by-step approach lays in tuning the signal at the intermediate points of the beams' paths (see Fig. 1). At each consecutive step, the interference intensity at the given point is matched with the theoretical value obtained through the breakdown of Eq. (13) from the main text. At the first two stages, we receive the signal reflected from the phase shifters $APS_2$ and $APS_1$ using the detectors $AD_1$ and $AD_2$, respectively. In turn, the last two stages involve the signals from the detectors $AD_3$ and $AD_4$. The alignment is performed via rotating the phase shifters (i.e., altering the optical path length) preceding the given point. By doing so, one changes the phases $x_i$ which in the end should be equal to $x_i^F$ given by Eq. (12) from the main text.

Let us now examine each step of the procedure in details.

*Step 1.–* Since $x_1$, $x_2$, $x_3$ and $x_4$ essentially determine the initial relative phases between the $|0\rangle$, $|1\rangle$ and $|2\rangle$ beams, we have a freedom in choosing $\phi$. This is easily seen by noticing from Eq. (11) of the main text that

$$p_i(\phi, \Delta x_1, \Delta x_2, \Delta x_3, \Delta x_4) = p_i(0, \Delta x_1 + \phi, \Delta x_2 + \phi, \Delta x_3, \Delta x_4 - \phi), \tag{1}$$

with $i = \{0, 1, 2\}$; here we denote $\Delta x_j = x_j - x_j^F$ $(j = \{1, 2, 3, 4\})$. Although the experimental value of $\phi$ (which shall be denoted $\phi^{real}$) set by $PS_\phi$ and $PS_{2\phi}$ is unknown, we can reassign $\phi$ to an arbitrary value. According to Eq. (1), in order to put $\phi = \phi_0$, we should renominate the target phases $x_i^F$ as follows:

$$\begin{aligned}
\widetilde{x}_1^F &= x_1^F - \phi^{real} + \phi_0;\\
\widetilde{x}_2^F &= x_2^F - \phi^{real} + \phi_0;\\
\widetilde{x}_3^F &= x_3^F;\\
\widetilde{x}_4^F &= x_4^F + \phi^{real} - \phi_0.
\end{aligned} \tag{2}$$

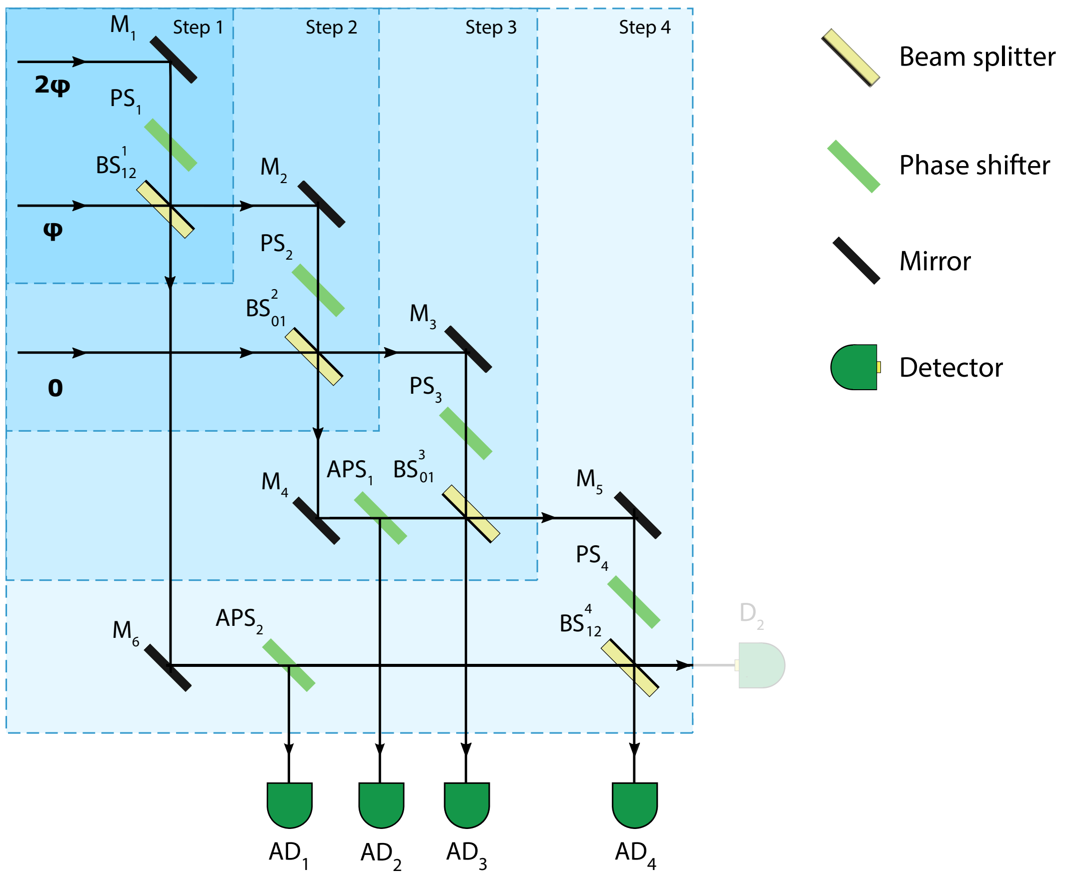


Figure 1: Four consecutive steps of the alignment procedure. At $i$th step, the output signal from the corresponding sector of the scheme (measured by the detector $AD_i$) is tuned to comply with the theoretical value calculated through the breakdown of Eq. (13) from the main text. The tuning is done through the alignment of $PS_i$.

Here and throughout the whole procedure we put $\phi = \pi/3$.

Using $\mathrm{AD}_1$ we measure the intensity of the $|1\rangle$ beam after it passes through $\mathrm{BS}^1_{12}$. This intensity may be regarded as the probability $\tilde{p}_1$ of finding the qutrit in the state $|1\rangle$ after the action of the first block of operators (denoted by $[\ldots]_1$) in Eq. (10) and may be written

$$\tilde{p}_1 = \sin^2(\chi_0)\cos^2(\chi_0)(1 - t_{\mathrm{ps}}t_{2\phi}t_{\mathrm{f}}(t_{\mathrm{ps}}t_{2\phi}t_{\mathrm{f}} + 2t_\phi \sin(\chi_0)\cos(\Delta x_1 + \phi)) - t_\phi^2 \sin^2(\chi_0)), \tag{3}$$

Our object is to set the value of $\Delta x_1$ to zero so that the measured signal would comply with the action of the first block in Eq. (13). Experimentally we achieve this by rotating $\mathrm{PS}_1$ and controlling the intensity on $\mathrm{AD}_1$. According to Eq. (3), the target intensity can be expressed in terms of the experimentally measurable values as $\tilde{p}_1 = \min_{x_1} \tilde{p}_1 + 0.25\,(\max_{x_1} \tilde{p}_1 - \min_{x_1} \tilde{p}_1)$. Fig. 2(a) shows the theoretical plot of the signal as function of $\Delta x_1$, where the dot marks the point to which we adjust $\mathrm{PS}_1$.

*Step 2.–* Using $\mathrm{AD}_2$ we measure the intensity of the $|0\rangle$ beam after it passes through $\mathrm{BS}^2_{01}$. Bearing in mind the second block of operators ($[\ldots]_2$), we write the corresponding probability $\tilde{p}_2$:

$$\begin{aligned}\tilde{p}_2 = \frac{1}{32}(\sin^2(2\chi_0)(8t_{\mathrm{ps}}\sin(2\chi_0)(t_f t_{\mathrm{ps}} t_{2\phi}(\sin(\Delta \mathrm{x}_2 + 2\phi) + t_{\mathrm{ps}}t_\phi \cos(\chi_0)\cos(\phi)) \\ + \sin(\Delta \mathrm{x}_2)t_\phi \sin(\chi_0)\cos(\phi)) + 4\cos(2\chi_0)(t_f^2 t_{\mathrm{ps}}^4 t_{2\phi}^2 - 1) \\ + 4t_f^2 t_{\mathrm{ps}}^4 t_{2\phi}^2 - t_{\mathrm{ps}}^2 t_\phi^2 \cos(4\chi_0) + t_{\mathrm{ps}}^2 t_\phi^2 + 4) + 64\cos(\Delta \mathrm{x}_2)t_{\mathrm{ps}}t_\phi \sin^4(\chi_0)\cos^3(\chi_0)\sin(\phi))\end{aligned} \tag{4}$$

The condition $\Delta x_2 = 0$ corresponds to a maximum of $\tilde{p}_2$ (see Fig. 2(b)).

*Step 3.–* Using $\mathrm{AD}_3$ we measure the intensity of the $|0\rangle$ beam after it passes through $\mathrm{BS}^3_{01}$. The corre-

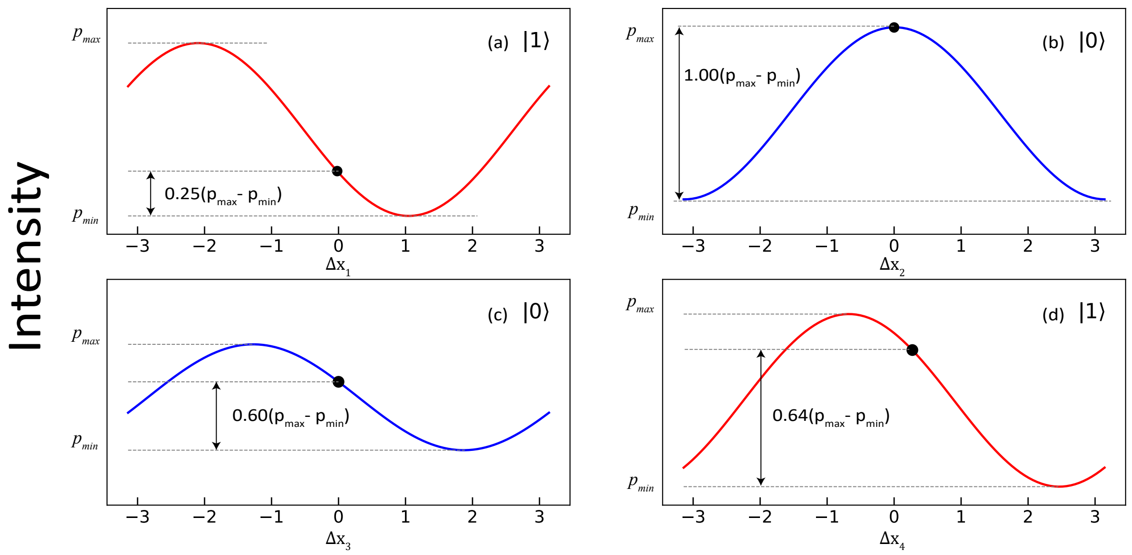


Figure 2: Alignment plots of intensities on various detectors as functions of $\Delta x_i$ $(i = \{1, 2, 3, 4\})$; $\Delta x_i$ is determined by the position of $\mathrm{PS}_i$. Black points correspond to the Fourier transform configuration. (a) Detector $\mathrm{AD}_1$; first step of alignment. (b) Detector $\mathrm{AD}_2$; second step. (c) Detector $\mathrm{AD}_3$; third step. (d) Detector $\mathrm{AD}_4$; fourth step.

sponding probability $\tilde{p}_3$ after the action of the third block operators $([\ldots]_3)$ is given by

$$\begin{aligned}
\tilde{p}_3 = \frac{1}{32}\Big(&\frac{32}{3} t_{\mathrm{ps}}^2 t_\phi \sin^4(\chi_0)\cos^3(\chi_0)\cos(\phi)(2(\sin(\Delta \mathrm{x}_3) + 2\sqrt{2}\cos(\Delta \mathrm{x}_3)) \\
&+ t_f t_{\mathrm{ps}} t_{2\phi}\sin(2\chi_0)(-4\sqrt{2}\sin(\Delta \mathrm{x}_3)t_{\mathrm{ps}} + 2\cos(\Delta \mathrm{x}_3)t_{\mathrm{ps}} + 3t_{\mathrm{ps}}^2 + 3)) \\
&+ \frac{1}{4}(\frac{8}{3} t_{\mathrm{ps}}\sin^4(2\chi_0)(\cos(\Delta \mathrm{x}_3) - 2\sqrt{2}\sin(\Delta \mathrm{x}_3))(2t_f^2 t_{\mathrm{ps}}^4 t_{2\phi}^2 - t_{\mathrm{ps}}^2 t_\phi^2\cos(2\chi_0) + t_{\mathrm{ps}}^2 t_\phi^2 - 2) \\
&+ \frac{1}{4}(2(t_{\mathrm{ps}}^2+1)\cos(8\chi_0)(2t_f^2 t_{\mathrm{ps}}^4 t_{2\phi}^2 + t_{\mathrm{ps}}^2 t_\phi^2 - 2) - 8(t_{\mathrm{ps}}^2+1)\cos(4\chi_0)(2t_f^2 t_{\mathrm{ps}}^4 t_{2\phi}^2 + t_{\mathrm{ps}}^2 t_\phi^2 + 2) \\
&+ 2(t_{\mathrm{ps}}^2+1)(6t_f^2 t_{\mathrm{ps}}^4 t_{2\phi}^2 + 3t_{\mathrm{ps}}^2 t_\phi^2 + 10) + t_{\mathrm{ps}}^2(t_{\mathrm{ps}}^2+1)t_\phi^2(-\cos(10\chi_0)) - 2\cos(2\chi_0)(t_{\mathrm{ps}}^2((t_{\mathrm{ps}}^2+1)t_\phi^2 - 8) + 8) \\
&+ \cos(6\chi_0)(t_{\mathrm{ps}}^2(3(t_{\mathrm{ps}}^2+1)t_\phi^2 - 16) + 16))) \\
&- 4t_f t_{\mathrm{ps}}^2 t_{2\phi}\sin^3(2\chi_0)\sin(2\phi)(\cos(2\chi_0)(\frac{2}{3}t_{\mathrm{ps}}(\cos(\Delta \mathrm{x}_3) - 2\sqrt{2}\sin(\Delta \mathrm{x}_3)) + t_{\mathrm{ps}}^2 + 1) + t_{\mathrm{ps}}^2 - 1) \\
&+ 8t_f t_{\mathrm{ps}}^3 t_{2\phi}\sin^3(2\chi_0)\cos(2\phi)\sin(2\tan^{-1}(\sqrt{2}) - \Delta \mathrm{x}_3) \\
&- 32t_{\mathrm{ps}} t_\phi\sin^4(\chi_0)\cos^3(\chi_0)\sin(\phi)(\cos(2\chi_0)(\frac{2}{3}t_{\mathrm{ps}}(\cos(\Delta \mathrm{x}_3) - 2\sqrt{2}\sin(\Delta \mathrm{x}_3)) + t_{\mathrm{ps}}^2 + 1) + t_{\mathrm{ps}}^2 - 1)\Big) \quad (5)
\end{aligned}$$

For $\Delta x_3 = 0$ we have $\tilde{p}_3 = \min_{x_3}\tilde{p}_3 + 0.60\,(\max_{x_3}\tilde{p}_3 - \min_{x_3}\tilde{p}_3)$ (see Fig. 2(c)).
*Step 4.–* Using $\mathrm{AD}_3$ we measure the intensity of the $|1\rangle$ beam after it passes through $\mathrm{BS}_{12}^4$. The corresponding probability $\tilde{p}_4$ after the action of the fourth block operators $([\ldots]_4)$ is given by

$$\begin{aligned}
\tilde{p}_4 = \frac{1}{2048}(&16\cos(8\chi_0)\sin^2(\chi_0)t_f^2 t_{2\phi}^2 t_{\mathrm{ps}}^8 + 48\sin^2(\chi_0)t_f^2 t_{2\phi}^2 t_{\mathrm{ps}}^8 - 16\cos(2\tan^{-1}(\sqrt{2}) - 8\chi_0)\sin^2(\chi_0)t_f^2 t_{2\phi}^2 t_{\mathrm{ps}}^7 \\
&- 64\cos(2\tan^{-1}(\sqrt{2}) - 6\chi_0)\sin^2(\chi_0)t_f^2 t_{2\phi}^2 t_{\mathrm{ps}}^7 - 64\cos(2\tan^{-1}(\sqrt{2}) - 4\chi_0)\sin^2(\chi_0)t_f^2 t_{2\phi}^2 t_{\mathrm{ps}}^7 \\
&+ 64\cos(2\tan^{-1}(\sqrt{2}) - 2\chi_0)\sin^2(\chi_0)t_f^2 t_{2\phi}^2 t_{\mathrm{ps}}^7 + 64\cos(2(\chi_0 + \tan^{-1}(\sqrt{2})))\sin^2(\chi_0)t_f^2 t_{2\phi}^2 t_{\mathrm{ps}}^7 \\
&- 64\cos(4\chi_0 + 2\tan^{-1}(\sqrt{2}))\sin^2(\chi_0)t_f^2 t_{2\phi}^2 t_{\mathrm{ps}}^7 - 64\cos(6\chi_0 + 2\tan^{-1}(\sqrt{2}))\sin^2(\chi_0)t_f^2 t_{2\phi}^2 t_{\mathrm{ps}}^7 \\
&- 16\cos(8\chi_0 + 2\tan^{-1}(\sqrt{2}))\sin^2(\chi_0)t_f^2 t_{2\phi}^2 t_{\mathrm{ps}}^7 - \frac{160}{3}\sin^2(\chi_0)t_f^2 t_{2\phi}^2 t_{\mathrm{ps}}^7 \\
&+ 48\cos(\phi)\sin(\chi_0)t_f t_\phi t_{2\phi}t_{\mathrm{ps}}^7 - 4\cos(\phi - 10\chi_0)\sin(\chi_0)t_f t_\phi t_{2\phi}t_{\mathrm{ps}}^7 + 8\cos(\phi - 8\chi_0)\sin(\chi_0)t_f t_\phi t_{2\phi}t_{\mathrm{ps}}^7 \\
&+ 12\cos(\phi - 6\chi_0)\sin(\chi_0)t_f t_\phi t_{2\phi}t_{\mathrm{ps}}^7 - 32\cos(\phi - 4\chi_0)\sin(\chi_0)t_f t_\phi t_{2\phi}t_{\mathrm{ps}}^7 - 8\cos(\phi - 2\chi_0)\sin(\chi_0)t_f t_\phi t_{2\phi}t_{\mathrm{ps}}^7 \\
&- 8\cos(\phi + 2\chi_0)\sin(\chi_0)t_f t_\phi t_{2\phi}t_{\mathrm{ps}}^7 - 32\cos(\phi + 4\chi_0)\sin(\chi_0)t_f t_\phi t_{2\phi}t_{\mathrm{ps}}^7 + 12\cos(\phi + 6\chi_0)\sin(\chi_0)t_f t_\phi t_{2\phi}t_{\mathrm{ps}}^7
\end{aligned}$$

$$+8\cos(\phi+8\chi_0)\sin(\chi_0)t_f t_\phi t_{2\phi}t_{\rm ps}^7-4\cos(\phi+10\chi_0)\sin(\chi_0)t_f t_\phi t_{2\phi}t_{\rm ps}^7+12\cos(6\chi_0)t_\phi^2 t_{\rm ps}^6+2\cos(8\chi_0)t_\phi^2 t_{\rm ps}^6$$
$$-4\cos(10\chi_0)t_\phi^2 t_{\rm ps}^6+\cos(12\chi_0)t_\phi^2 t_{\rm ps}^6+128\cos(6\chi_0)\sin^2(\chi_0)t_f^2 t_{2\phi}^2 t_{\rm ps}^6+16\cos(8\chi_0)\sin^2(\chi_0)t_f^2 t_{2\phi}^2 t_{\rm ps}^6$$
$$+560\sin^2(\chi_0)t_f^2 t_{2\phi}^2 t_{\rm ps}^6-512\cos(\phi)\cos(\chi_0)\sin(\phi)\sin^3(\chi_0)t_f t_{2\phi}t_{\rm ps}^6+64\cos(\chi_0)\sin(2\phi-6\chi_0)\sin^3(\chi_0)t_f t_{2\phi}t_{\rm ps}^6$$
$$+128\cos(\chi_0)\sin(2\phi-4\chi_0)\sin^3(\chi_0)t_f t_{2\phi}t_{\rm ps}^6-64\cos(\chi_0)\sin(2\phi-2\chi_0)\sin^3(\chi_0)t_f t_{2\phi}t_{\rm ps}^6$$
$$-64\cos(\chi_0)\sin^3(\chi_0)\sin(2(\phi+\chi_0))t_f t_{2\phi}t_{\rm ps}^6+128\cos(\chi_0)\sin^3(\chi_0)\sin(2\phi+4\chi_0)t_f t_{2\phi}t_{\rm ps}^6$$
$$+64\cos(\chi_0)\sin^3(\chi_0)\sin(2\phi+6\chi_0)t_f t_{2\phi}t_{\rm ps}^6+48\cos(\phi-2\tan^{-1}(\sqrt{2}))\sin(\chi_0)t_f t_\phi t_{2\phi}t_{\rm ps}^6$$
$$+48\cos(\phi+2\tan^{-1}(\sqrt{2}))\sin(\chi_0)t_f t_\phi t_{2\phi}t_{\rm ps}^6+4\cos(\phi-10\chi_0+2\tan^{-1}(\sqrt{2}))\sin(\chi_0)t_f t_\phi t_{2\phi}t_{\rm ps}^6$$
$$+8\cos(\phi-8\chi_0-2\tan^{-1}(\sqrt{2}))\sin(\chi_0)t_f t_\phi t_{2\phi}t_{\rm ps}^6+8\cos(\phi-8\chi_0+2\tan^{-1}(\sqrt{2}))\sin(\chi_0)t_f t_\phi t_{2\phi}t_{\rm ps}^6$$
$$-12\cos(\phi-6\chi_0-2\tan^{-1}(\sqrt{2}))\sin(\chi_0)t_f t_\phi t_{2\phi}t_{\rm ps}^6-12\cos(\phi-6\chi_0+2\tan^{-1}(\sqrt{2}))\sin(\chi_0)t_f t_\phi t_{2\phi}t_{\rm ps}^6$$
$$-32\cos(\phi-4\chi_0-2\tan^{-1}(\sqrt{2}))\sin(\chi_0)t_f t_\phi t_{2\phi}t_{\rm ps}^6-32\cos(\phi-4\chi_0+2\tan^{-1}(\sqrt{2}))\sin(\chi_0)t_f t_\phi t_{2\phi}t_{\rm ps}^6$$
$$+8\cos(\phi-2\chi_0+2\tan^{-1}(\sqrt{2}))\sin(\chi_0)t_f t_\phi t_{2\phi}t_{\rm ps}^6+8\cos(\phi+2\chi_0-2\tan^{-1}(\sqrt{2}))\sin(\chi_0)t_f t_\phi t_{2\phi}t_{\rm ps}^6$$
$$-32\cos(\phi+4\chi_0-2\tan^{-1}(\sqrt{2}))\sin(\chi_0)t_f t_\phi t_{2\phi}t_{\rm ps}^6-32\cos(\phi+4\chi_0+2\tan^{-1}(\sqrt{2}))\sin(\chi_0)t_f t_\phi t_{2\phi}t_{\rm ps}^6$$
$$-12\cos(\phi+6\chi_0-2\tan^{-1}(\sqrt{2}))\sin(\chi_0)t_f t_\phi t_{2\phi}t_{\rm ps}^6-12\cos(\phi+6\chi_0+2\tan^{-1}(\sqrt{2}))\sin(\chi_0)t_f t_\phi t_{2\phi}t_{\rm ps}^6$$
$$+8\cos(\phi+8\chi_0-2\tan^{-1}(\sqrt{2}))\sin(\chi_0)t_f t_\phi t_{2\phi}t_{\rm ps}^6+8\cos(\phi+8\chi_0+2\tan^{-1}(\sqrt{2}))\sin(\chi_0)t_f t_\phi t_{2\phi}t_{\rm ps}^6$$
$$+4\cos(\phi+10\chi_0-2\tan^{-1}(\sqrt{2}))\sin(\chi_0)t_f t_\phi t_{2\phi}t_{\rm ps}^6+4\cos(\phi+10\chi_0+2\tan^{-1}(\sqrt{2}))\sin(\chi_0)t_f t_\phi t_{2\phi}t_{\rm ps}^6$$
$$+8\cos(\phi-2(\chi_0+\tan^{-1}(\sqrt{2})))\sin(\chi_0)t_f t_\phi t_{2\phi}t_{\rm ps}^6+8\cos(\phi+2(\chi_0+\tan^{-1}(\sqrt{2})))\sin(\chi_0)t_f t_\phi t_{2\phi}t_{\rm ps}^6$$
$$+4\cos(\phi-2(5\chi_0+\tan^{-1}(\sqrt{2})))\sin(\chi_0)t_f t_\phi t_{2\phi}t_{\rm ps}^6-\cos(2\tan^{-1}(\sqrt{2})-12\chi_0)t_\phi^2 t_{\rm ps}^5$$
$$+6\cos(2\tan^{-1}(\sqrt{2})-8\chi_0)t_\phi^2 t_{\rm ps}^5-15\cos(2\tan^{-1}(\sqrt{2})-4\chi_0)t_\phi^2 t_{\rm ps}^5-15\cos(4\chi_0+2\tan^{-1}(\sqrt{2}))t_\phi^2 t_{\rm ps}^5$$
$$-\cos(2(6\chi_0+\tan^{-1}(\sqrt{2})))t_\phi^2 t_{\rm ps}^5+6\cos(8\chi_0+2\tan^{-1}(\sqrt{2}))t_\phi^2 t_{\rm ps}^5+256\sin(\tan^{-1}(\sqrt{2})-\Delta{\rm x}_4)\sin^2(\chi_0)t_f^2 t_{2\phi}^2 t_{\rm ps}^5$$
$$-64\sin(-\Delta{\rm x}_4-6\chi_0+\tan^{-1}(\sqrt{2}))\sin^2(\chi_0)t_f^2 t_{2\phi}^2 t_{\rm ps}^5-128\sin(-\Delta{\rm x}_4-4\chi_0+\tan^{-1}(\sqrt{2}))\sin^2(\chi_0)t_f^2 t_{2\phi}^2 t_{\rm ps}^5$$
$$+64\sin(-\Delta{\rm x}_4-2\chi_0+\tan^{-1}(\sqrt{2}))\sin^2(\chi_0)t_f^2 t_{2\phi}^2 t_{\rm ps}^5+64\sin^2(\chi_0)\sin(-\Delta{\rm x}_4+2\chi_0+\tan^{-1}(\sqrt{2}))t_f^2 t_{2\phi}^2 t_{\rm ps}^5$$
$$-128\sin^2(\chi_0)\sin(-\Delta{\rm x}_4+4\chi_0+\tan^{-1}(\sqrt{2}))t_f^2 t_{2\phi}^2 t_{\rm ps}^5-64\sin^2(\chi_0)\sin(-\Delta{\rm x}_4+6\chi_0+\tan^{-1}(\sqrt{2}))t_f^2 t_{2\phi}^2 t_{\rm ps}^5$$
$$-96\cos(\chi_0)\sin(\phi)\sin^2(\chi_0)t_\phi t_{\rm ps}^5+64\cos(\chi_0)\sin(\phi-4\chi_0)\sin^2(\chi_0)t_\phi t_{\rm ps}^5-8\sin(\phi-8\chi_0)\sin(\chi_0)\sin(2\chi_0)t_\phi t_{\rm ps}^5$$
$$+64\cos(\chi_0)\sin^2(\chi_0)\sin(\phi+4\chi_0)t_\phi t_{\rm ps}^5-8\sin(\chi_0)\sin(2\chi_0)\sin(\phi+8\chi_0)t_\phi t_{\rm ps}^5$$
$$+256\cos(\chi_0)\sin(2\phi-2\tan^{-1}(\sqrt{2}))\sin^3(\chi_0)t_f t_{2\phi}t_{\rm ps}^5-1280\cos(\chi_0)\sin(2(\phi+\tan^{-1}(\sqrt{2})))\sin^3(\chi_0)t_f t_{2\phi}t_{\rm ps}^5$$
$$-64\cos(\chi_0)\sin(2\phi-6\chi_0-2\tan^{-1}(\sqrt{2}))\sin^3(\chi_0)t_f t_{2\phi}t_{\rm ps}^5$$
$$-128\cos(\chi_0)\sin(2\phi-4\chi_0-2\tan^{-1}(\sqrt{2}))\sin^3(\chi_0)t_f t_{2\phi}t_{\rm ps}^5$$
$$-64\cos(\chi_0)\sin(2(\phi-3\chi_0+\tan^{-1}(\sqrt{2})))\sin^3(\chi_0)t_f t_{2\phi}t_{\rm ps}^5$$
$$+64\cos(\chi_0)\sin(2\phi-2\chi_0-2\tan^{-1}(\sqrt{2}))\sin^3(\chi_0)t_f t_{2\phi}t_{\rm ps}^5$$
$$-384\cos(\chi_0)\sin(2(\phi-2\chi_0+\tan^{-1}(\sqrt{2})))\sin^3(\chi_0)t_f t_{2\phi}t_{\rm ps}^5$$
$$-960\cos(\chi_0)\sin(2(\phi-\chi_0+\tan^{-1}(\sqrt{2})))\sin^3(\chi_0)t_f t_{2\phi}t_{\rm ps}^5$$
$$+64\cos(\chi_0)\sin^3(\chi_0)\sin(2(\phi+\chi_0-\tan^{-1}(\sqrt{2})))t_f t_{2\phi}t_{\rm ps}^5$$
$$-960\cos(\chi_0)\sin^3(\chi_0)\sin(2(\phi+\chi_0+\tan^{-1}(\sqrt{2})))t_f t_{2\phi}t_{\rm ps}^5$$
$$-384\cos(\chi_0)\sin^3(\chi_0)\sin(2(\phi+2\chi_0+\tan^{-1}(\sqrt{2})))t_f t_{2\phi}t_{\rm ps}^5$$
$$-64\cos(\chi_0)\sin^3(\chi_0)\sin(2(\phi+3\chi_0+\tan^{-1}(\sqrt{2})))t_f t_{2\phi}t_{\rm ps}^5$$
$$-128\cos(\chi_0)\sin^3(\chi_0)\sin(2\phi+4\chi_0-2\tan^{-1}(\sqrt{2}))t_f t_{2\phi}t_{\rm ps}^5$$
$$-64\cos(\chi_0)\sin^3(\chi_0)\sin(2\phi+6\chi_0-2\tan^{-1}(\sqrt{2}))t_f t_{2\phi}t_{\rm ps}^5+112\cos(\phi)\sin(\chi_0)t_f t_\phi t_{2\phi}t_{\rm ps}^5$$
$$-4\cos(\phi-10\chi_0)\sin(\chi_0)t_f t_\phi t_{2\phi}t_{\rm ps}^5-24\cos(\phi-8\chi_0)\sin(\chi_0)t_f t_\phi t_{2\phi}t_{\rm ps}^5-52\cos(\phi-6\chi_0)\sin(\chi_0)t_f t_\phi t_{2\phi}t_{\rm ps}^5$$
$$-32\cos(\phi-4\chi_0)\sin(\chi_0)t_f t_\phi t_{2\phi}t_{\rm ps}^5+56\cos(\phi-2\chi_0)\sin(\chi_0)t_f t_\phi t_{2\phi}t_{\rm ps}^5+56\cos(\phi+2\chi_0)\sin(\chi_0)t_f t_\phi t_{2\phi}t_{\rm ps}^5$$
$$-32\cos(\phi+4\chi_0)\sin(\chi_0)t_f t_\phi t_{2\phi}t_{\rm ps}^5-52\cos(\phi+6\chi_0)\sin(\chi_0)t_f t_\phi t_{2\phi}t_{\rm ps}^5-24\cos(\phi+8\chi_0)\sin(\chi_0)t_f t_\phi t_{2\phi}t_{\rm ps}^5$$
$$-4\cos(\phi+10\chi_0)\sin(\chi_0)t_f t_\phi t_{2\phi}t_{\rm ps}^5-12\cos(6\chi_0)t_\phi^2 t_{\rm ps}^4+2\cos(8\chi_0)t_\phi^2 t_{\rm ps}^4+4\cos(10\chi_0)t_\phi^2 t_{\rm ps}^4+\cos(12\chi_0)t_\phi^2 t_{\rm ps}^4$$

$$
\begin{aligned}
&- 1280\sin(\Delta\mathrm{x}_4 + \tan^{-1}(\sqrt{2}))\sin^2(\chi_0)t_f^2 t_{2\phi}^2 t_{\mathrm{ps}}^4 - 64\sin(\Delta\mathrm{x}_4 - 6\chi_0 + \tan^{-1}(\sqrt{2}))\sin^2(\chi_0)t_f^2 t_{2\phi}^2 t_{\mathrm{ps}}^4 \\
&- 384\sin(\Delta\mathrm{x}_4 - 4\chi_0 + \tan^{-1}(\sqrt{2}))\sin^2(\chi_0)t_f^2 t_{2\phi}^2 t_{\mathrm{ps}}^4 - 960\sin(\Delta\mathrm{x}_4 - 2\chi_0 + \tan^{-1}(\sqrt{2}))\sin^2(\chi_0)t_f^2 t_{2\phi}^2 t_{\mathrm{ps}}^4 \\
&- 960\sin^2(\chi_0)\sin(\Delta\mathrm{x}_4 + 2\chi_0 + \tan^{-1}(\sqrt{2}))t_f^2 t_{2\phi}^2 t_{\mathrm{ps}}^4 - 384\sin^2(\chi_0)\sin(\Delta\mathrm{x}_4 + 4\chi_0 + \tan^{-1}(\sqrt{2}))t_f^2 t_{2\phi}^2 t_{\mathrm{ps}}^4 \\
&- 64\sin^2(\chi_0)\sin(\Delta\mathrm{x}_4 + 6\chi_0 + \tan^{-1}(\sqrt{2}))t_f^2 t_{2\phi}^2 t_{\mathrm{ps}}^4 - 12\cos(6\chi_0)t_{\mathrm{ps}}^4 + 8\cos(8\chi_0)t_{\mathrm{ps}}^4 + 4\cos(10\chi_0)t_{\mathrm{ps}}^4 \\
&+ 96\cos(\chi_0)\sin(\phi - 2\tan^{-1}(\sqrt{2}))\sin^2(\chi_0)t_\phi t_{\mathrm{ps}}^4 + 64\cos(\chi_0)\sin(\phi - 6\chi_0 + 2\tan^{-1}(\sqrt{2}))\sin^2(\chi_0)t_\phi t_{\mathrm{ps}}^4 \\
&- 64\cos(\chi_0)\sin(\phi - 4\chi_0 - 2\tan^{-1}(\sqrt{2}))\sin^2(\chi_0)t_\phi t_{\mathrm{ps}}^4 + 64\cos(\chi_0)\sin(\phi - 4\chi_0 + 2\tan^{-1}(\sqrt{2}))\sin^2(\chi_0)t_\phi t_{\mathrm{ps}}^4 \\
&- 64\cos(\chi_0)\sin(\phi - 2\chi_0 + 2\tan^{-1}(\sqrt{2}))\sin^2(\chi_0)t_\phi t_{\mathrm{ps}}^4 - 80\sin(\phi + 2\tan^{-1}(\sqrt{2}))\sin(\chi_0)\sin(2\chi_0)t_\phi t_{\mathrm{ps}}^4 \\
&+ 8\sin(\phi - 8\chi_0 - 2\tan^{-1}(\sqrt{2}))\sin(\chi_0)\sin(2\chi_0)t_\phi t_{\mathrm{ps}}^4 + 8\sin(\phi - 8\chi_0 + 2\tan^{-1}(\sqrt{2}))\sin(\chi_0)\sin(2\chi_0)t_\phi t_{\mathrm{ps}}^4 \\
&- 64\cos(\chi_0)\sin^2(\chi_0)\sin(\phi + 4\chi_0 - 2\tan^{-1}(\sqrt{2}))t_\phi t_{\mathrm{ps}}^4 + 64\cos(\chi_0)\sin^2(\chi_0)\sin(\phi + 4\chi_0 + 2\tan^{-1}(\sqrt{2}))t_\phi t_{\mathrm{ps}}^4 \\
&+ 64\cos(\chi_0)\sin^2(\chi_0)\sin(\phi + 6\chi_0 + 2\tan^{-1}(\sqrt{2}))t_\phi t_{\mathrm{ps}}^4 + 8\sin(\chi_0)\sin(2\chi_0)\sin(\phi + 8\chi_0 - 2\tan^{-1}(\sqrt{2}))t_\phi t_{\mathrm{ps}}^4 \\
&+ 8\sin(\chi_0)\sin(2\chi_0)\sin(\phi + 8\chi_0 + 2\tan^{-1}(\sqrt{2}))t_\phi t_{\mathrm{ps}}^4 - 64\cos(\chi_0)\sin^2(\chi_0)\sin(\phi + 2(\chi_0 + \tan^{-1}(\sqrt{2})))t_\phi t_{\mathrm{ps}}^4 \\
&+ 2560\cos(\phi)\cos(\chi_0)\sin(\phi)\sin^3(\chi_0)t_f t_{2\phi} t_{\mathrm{ps}}^4 + 64\cos(\chi_0)\sin(2\phi - 6\chi_0)\sin^3(\chi_0)t_f t_{2\phi} t_{\mathrm{ps}}^4 \\
&+ 384\cos(\chi_0)\sin(2\phi - 4\chi_0)\sin^3(\chi_0)t_f t_{2\phi} t_{\mathrm{ps}}^4 + 960\cos(\chi_0)\sin(2\phi - 2\chi_0)\sin^3(\chi_0)t_f t_{2\phi} t_{\mathrm{ps}}^4 \\
&+ 960\cos(\chi_0)\sin^3(\chi_0)\sin(2(\phi + \chi_0))t_f t_{2\phi} t_{\mathrm{ps}}^4 + 384\cos(\chi_0)\sin^3(\chi_0)\sin(2\phi + 4\chi_0)t_f t_{2\phi} t_{\mathrm{ps}}^4 \\
&+ 64\cos(\chi_0)\sin^3(\chi_0)\sin(2\phi + 6\chi_0)t_f t_{2\phi} t_{\mathrm{ps}}^4 + 96\sin(\phi - \Delta\mathrm{x}_4 + \tan^{-1}(\sqrt{2}))\sin(\chi_0)t_f t_\phi t_{2\phi} t_{\mathrm{ps}}^4 \\
&+ 160\sin(\phi + \Delta\mathrm{x}_4 - \tan^{-1}(\sqrt{2}))\sin(\chi_0)t_f t_\phi t_{2\phi} t_{\mathrm{ps}}^4 + 16\sin(\phi - \Delta\mathrm{x}_4 - 8\chi_0 + \tan^{-1}(\sqrt{2}))\sin(\chi_0)t_f t_\phi t_{2\phi} t_{\mathrm{ps}}^4 \\
&- 16\sin(\phi + \Delta\mathrm{x}_4 - 8\chi_0 - \tan^{-1}(\sqrt{2}))\sin(\chi_0)t_f t_\phi t_{2\phi} t_{\mathrm{ps}}^4 + 64\sin(\phi + \Delta\mathrm{x}_4 - 6\chi_0 - \tan^{-1}(\sqrt{2}))\sin(\chi_0)t_f t_\phi t_{2\phi} t_{\mathrm{ps}}^4 \\
&- 64\sin(\phi - \Delta\mathrm{x}_4 - 4\chi_0 + \tan^{-1}(\sqrt{2}))\sin(\chi_0)t_f t_\phi t_{2\phi} t_{\mathrm{ps}}^4 - 64\sin(\phi + \Delta\mathrm{x}_4 - 4\chi_0 - \tan^{-1}(\sqrt{2}))\sin(\chi_0)t_f t_\phi t_{2\phi} t_{\mathrm{ps}}^4 \\
&- 64\sin(\phi + \Delta\mathrm{x}_4 - 2\chi_0 - \tan^{-1}(\sqrt{2}))\sin(\chi_0)t_f t_\phi t_{2\phi} t_{\mathrm{ps}}^4 - 64\sin(\chi_0)\sin(\phi + \Delta\mathrm{x}_4 + 2\chi_0 - \tan^{-1}(\sqrt{2}))t_f t_\phi t_{2\phi} t_{\mathrm{ps}}^4 \\
&- 64\sin(\chi_0)\sin(\phi - \Delta\mathrm{x}_4 + 4\chi_0 + \tan^{-1}(\sqrt{2}))t_f t_\phi t_{2\phi} t_{\mathrm{ps}}^4 - 64\sin(\chi_0)\sin(\phi + \Delta\mathrm{x}_4 + 4\chi_0 - \tan^{-1}(\sqrt{2}))t_f t_\phi t_{2\phi} t_{\mathrm{ps}}^4 \\
&+ 64\sin(\chi_0)\sin(\phi + \Delta\mathrm{x}_4 + 6\chi_0 - \tan^{-1}(\sqrt{2}))t_f t_\phi t_{2\phi} t_{\mathrm{ps}}^4 + 16\sin(\chi_0)\sin(\phi - \Delta\mathrm{x}_4 + 8\chi_0 + \tan^{-1}(\sqrt{2}))t_f t_\phi t_{2\phi} t_{\mathrm{ps}}^4 \\
&- 16\sin(\chi_0)\sin(\phi + \Delta\mathrm{x}_4 + 8\chi_0 - \tan^{-1}(\sqrt{2}))t_f t_\phi t_{2\phi} t_{\mathrm{ps}}^4 - 112\sin(\tan^{-1}(\sqrt{2}) - \Delta\mathrm{x}_4)t_\phi^2 t_{\mathrm{ps}}^3 \\
&- 4\sin(-\Delta\mathrm{x}_4 - 10\chi_0 + \tan^{-1}(\sqrt{2}))t_\phi^2 t_{\mathrm{ps}}^3 + 24\sin(-\Delta\mathrm{x}_4 - 8\chi_0 + \tan^{-1}(\sqrt{2}))t_\phi^2 t_{\mathrm{ps}}^3 \\
&- 52\sin(-\Delta\mathrm{x}_4 - 6\chi_0 + \tan^{-1}(\sqrt{2}))t_\phi^2 t_{\mathrm{ps}}^3 + 32\sin(-\Delta\mathrm{x}_4 - 4\chi_0 + \tan^{-1}(\sqrt{2}))t_\phi^2 t_{\mathrm{ps}}^3 \\
&+ 56\sin(-\Delta\mathrm{x}_4 - 2\chi_0 + \tan^{-1}(\sqrt{2}))t_\phi^2 t_{\mathrm{ps}}^3 + 56\sin(-\Delta\mathrm{x}_4 + 2\chi_0 + \tan^{-1}(\sqrt{2}))t_\phi^2 t_{\mathrm{ps}}^3 \\
&+ 32\sin(-\Delta\mathrm{x}_4 + 4\chi_0 + \tan^{-1}(\sqrt{2}))t_\phi^2 t_{\mathrm{ps}}^3 - 52\sin(-\Delta\mathrm{x}_4 + 6\chi_0 + \tan^{-1}(\sqrt{2}))t_\phi^2 t_{\mathrm{ps}}^3 \\
&+ 24\sin(-\Delta\mathrm{x}_4 + 8\chi_0 + \tan^{-1}(\sqrt{2}))t_\phi^2 t_{\mathrm{ps}}^3 - 4\sin(-\Delta\mathrm{x}_4 + 10\chi_0 + \tan^{-1}(\sqrt{2}))t_\phi^2 t_{\mathrm{ps}}^3 - 4\cos(2\tan^{-1}(\sqrt{2}) - 10\chi_0)t_{\mathrm{ps}}^3 \\
&- 8\cos(2\tan^{-1}(\sqrt{2}) - 8\chi_0)t_{\mathrm{ps}}^3 + 12\cos(2\tan^{-1}(\sqrt{2}) - 6\chi_0)t_{\mathrm{ps}}^3 + 32\cos(2\tan^{-1}(\sqrt{2}) - 4\chi_0)t_{\mathrm{ps}}^3 \\
&- 8\cos(2\tan^{-1}(\sqrt{2}) - 2\chi_0)t_{\mathrm{ps}}^3 - 8\cos(2(\chi_0 + \tan^{-1}(\sqrt{2})))t_{\mathrm{ps}}^3 + 32\cos(4\chi_0 + 2\tan^{-1}(\sqrt{2}))t_{\mathrm{ps}}^3 \\
&- 4\cos(2(5\chi_0 + \tan^{-1}(\sqrt{2})))t_{\mathrm{ps}}^3 + 12\cos(6\chi_0 + 2\tan^{-1}(\sqrt{2}))t_{\mathrm{ps}}^3 - 8\cos(8\chi_0 + 2\tan^{-1}(\sqrt{2}))t_{\mathrm{ps}}^3 \\
&- 64\cos(\chi_0)\sin(\phi - 6\chi_0)\sin^2(\chi_0)t_\phi t_{\mathrm{ps}}^3 - 64\cos(\chi_0)\sin(\phi - 4\chi_0)\sin^2(\chi_0)t_\phi t_{\mathrm{ps}}^3 + 64\cos(\chi_0)\sin(\phi - 2\chi_0)\sin^2(\chi_0)t_\phi t_{\mathrm{ps}}^3 \\
&+ 80\sin(\phi)\sin(\chi_0)\sin(2\chi_0)t_\phi t_{\mathrm{ps}}^3 - 8\sin(\phi - 8\chi_0)\sin(\chi_0)\sin(2\chi_0)t_\phi t_{\mathrm{ps}}^3 + 64\cos(\chi_0)\sin^2(\chi_0)\sin(\phi + 2\chi_0)t_\phi t_{\mathrm{ps}}^3 \\
&- 64\cos(\chi_0)\sin^2(\chi_0)\sin(\phi + 4\chi_0)t_\phi t_{\mathrm{ps}}^3 - 64\cos(\chi_0)\sin^2(\chi_0)\sin(\phi + 6\chi_0)t_\phi t_{\mathrm{ps}}^3 - 8\sin(\chi_0)\sin(2\chi_0)\sin(\phi + 8\chi_0)t_\phi t_{\mathrm{ps}}^3 \\
&+ 1536\cos(2\phi - \Delta\mathrm{x}_4 + \tan^{-1}(\sqrt{2}))\cos(\chi_0)\sin^3(\chi_0)t_f t_{2\phi} t_{\mathrm{ps}}^3 \\
&+ 256\cos(2\phi - \Delta\mathrm{x}_4 - 4\chi_0 + \tan^{-1}(\sqrt{2}))\cos(\chi_0)\sin^3(\chi_0)t_f t_{2\phi} t_{\mathrm{ps}}^3 \\
&+ 1024\cos(2\phi - \Delta\mathrm{x}_4 - 2\chi_0 + \tan^{-1}(\sqrt{2}))\cos(\chi_0)\sin^3(\chi_0)t_f t_{2\phi} t_{\mathrm{ps}}^3 \\
&+ 256\cos(\chi_0)\cos(2\phi - \Delta\mathrm{x}_4 + 4\chi_0 + \tan^{-1}(\sqrt{2}))\sin^3(\chi_0)t_f t_{2\phi} t_{\mathrm{ps}}^3 \\
&+ 1024\cos(\chi_0)\cos(-\Delta\mathrm{x}_4 + 2(\phi + \chi_0) + \tan^{-1}(\sqrt{2}))\sin^3(\chi_0)t_f t_{2\phi} t_{\mathrm{ps}}^3 \\
&+ 160\sin(\phi - \Delta\mathrm{x}_4 - \tan^{-1}(\sqrt{2}))\sin(\chi_0)t_f t_\phi t_{2\phi} t_{\mathrm{ps}}^3 + 96\sin(\phi + \Delta\mathrm{x}_4 + \tan^{-1}(\sqrt{2}))\sin(\chi_0)t_f t_\phi t_{2\phi} t_{\mathrm{ps}}^3 \\
&- 16\sin(\phi - \Delta\mathrm{x}_4 - 8\chi_0 - \tan^{-1}(\sqrt{2}))\sin(\chi_0)t_f t_\phi t_{2\phi} t_{\mathrm{ps}}^3 + 16\sin(\phi + \Delta\mathrm{x}_4 - 8\chi_0 + \tan^{-1}(\sqrt{2}))\sin(\chi_0)t_f t_\phi t_{2\phi} t_{\mathrm{ps}}^3
\end{aligned}
$$

$$
\begin{aligned}
&-64\sin(\phi-\Delta\mathrm{x}_4-6\chi_0-\tan^{-1}(\sqrt{2}))\sin(\chi_0)t_f t_\phi t_{2\phi}t_{\mathrm{ps}}^3-64\sin(\phi-\Delta\mathrm{x}_4-4\chi_0-\tan^{-1}(\sqrt{2}))\sin(\chi_0)t_f t_\phi t_{2\phi}t_{\mathrm{ps}}^3\\
&-64\sin(\phi+\Delta\mathrm{x}_4-4\chi_0+\tan^{-1}(\sqrt{2}))\sin(\chi_0)t_f t_\phi t_{2\phi}t_{\mathrm{ps}}^3+64\sin(\phi-\Delta\mathrm{x}_4-2\chi_0-\tan^{-1}(\sqrt{2}))\sin(\chi_0)t_f t_\phi t_{2\phi}t_{\mathrm{ps}}^3\\
&+64\sin(\chi_0)\sin(\phi-\Delta\mathrm{x}_4+2\chi_0-\tan^{-1}(\sqrt{2}))t_f t_\phi t_{2\phi}t_{\mathrm{ps}}^3-64\sin(\chi_0)\sin(\phi-\Delta\mathrm{x}_4+4\chi_0-\tan^{-1}(\sqrt{2}))t_f t_\phi t_{2\phi}t_{\mathrm{ps}}^3\\
&-64\sin(\chi_0)\sin(\phi+\Delta\mathrm{x}_4+4\chi_0+\tan^{-1}(\sqrt{2}))t_f t_\phi t_{2\phi}t_{\mathrm{ps}}^3-64\sin(\chi_0)\sin(\phi-\Delta\mathrm{x}_4+6\chi_0-\tan^{-1}(\sqrt{2}))t_f t_\phi t_{2\phi}t_{\mathrm{ps}}^3\\
&-16\sin(\chi_0)\sin(\phi-\Delta\mathrm{x}_4+8\chi_0-\tan^{-1}(\sqrt{2}))t_f t_\phi t_{2\phi}t_{\mathrm{ps}}^3+16\sin(\chi_0)\sin(\phi+\Delta\mathrm{x}_4+8\chi_0+\tan^{-1}(\sqrt{2}))t_f t_\phi t_{2\phi}t_{\mathrm{ps}}^3\\
&+48\sin(\Delta\mathrm{x}_4+\tan^{-1}(\sqrt{2}))t_\phi^2 t_{\mathrm{ps}}^2-4\sin(\Delta\mathrm{x}_4-10\chi_0+\tan^{-1}(\sqrt{2}))t_\phi^2 t_{\mathrm{ps}}^2+8\sin(\Delta\mathrm{x}_4-8\chi_0+\tan^{-1}(\sqrt{2}))t_\phi^2 t_{\mathrm{ps}}^2\\
&+12\sin(\Delta\mathrm{x}_4-6\chi_0+\tan^{-1}(\sqrt{2}))t_\phi^2 t_{\mathrm{ps}}^2-32\sin(\Delta\mathrm{x}_4-4\chi_0+\tan^{-1}(\sqrt{2}))t_\phi^2 t_{\mathrm{ps}}^2\\
&-8\sin(\Delta\mathrm{x}_4-2\chi_0+\tan^{-1}(\sqrt{2}))t_\phi^2 t_{\mathrm{ps}}^2-8\sin(\Delta\mathrm{x}_4+2\chi_0+\tan^{-1}(\sqrt{2}))t_\phi^2 t_{\mathrm{ps}}^2\\
&-32\sin(\Delta\mathrm{x}_4+4\chi_0+\tan^{-1}(\sqrt{2}))t_\phi^2 t_{\mathrm{ps}}^2+12\sin(\Delta\mathrm{x}_4+6\chi_0+\tan^{-1}(\sqrt{2}))t_\phi^2 t_{\mathrm{ps}}^2\\
&+8\sin(\Delta\mathrm{x}_4+8\chi_0+\tan^{-1}(\sqrt{2}))t_\phi^2 t_{\mathrm{ps}}^2-4\sin(\Delta\mathrm{x}_4+10\chi_0+\tan^{-1}(\sqrt{2}))t_\phi^2 t_{\mathrm{ps}}^2+768\sin^2(\chi_0)t_f^2 t_{2\phi}^2 t_{\mathrm{ps}}^2\\
&-12\cos(6\chi_0)t_{\mathrm{ps}}^2+8\cos(8\chi_0)t_{\mathrm{ps}}^2+4\cos(10\chi_0)t_{\mathrm{ps}}^2+8(t_{\mathrm{ps}}(3t_{\mathrm{ps}}+2)+3)t_{\mathrm{ps}}^2\\
&-256\cos(\phi-\Delta\mathrm{x}_4+\tan^{-1}(\sqrt{2}))\cos(\chi_0)\sin^2(\chi_0)t_\phi t_{\mathrm{ps}}^2-64\cos(\phi-\Delta\mathrm{x}_4-6\chi_0+\tan^{-1}(\sqrt{2}))\cos(\chi_0)\sin^2(\chi_0)t_\phi t_{\mathrm{ps}}^2\\
&+128\cos(\phi-\Delta\mathrm{x}_4-4\chi_0+\tan^{-1}(\sqrt{2}))\cos(\chi_0)\sin^2(\chi_0)t_\phi t_{\mathrm{ps}}^2\\
&+64\cos(\phi-\Delta\mathrm{x}_4-2\chi_0+\tan^{-1}(\sqrt{2}))\cos(\chi_0)\sin^2(\chi_0)t_\phi t_{\mathrm{ps}}^2\\
&+64\cos(\chi_0)\cos(\phi-\Delta\mathrm{x}_4+2\chi_0+\tan^{-1}(\sqrt{2}))\sin^2(\chi_0)t_\phi t_{\mathrm{ps}}^2\\
&+128\cos(\chi_0)\cos(\phi-\Delta\mathrm{x}_4+4\chi_0+\tan^{-1}(\sqrt{2}))\sin^2(\chi_0)t_\phi t_{\mathrm{ps}}^2\\
&-64\cos(\chi_0)\cos(\phi-\Delta\mathrm{x}_4+6\chi_0+\tan^{-1}(\sqrt{2}))\sin^2(\chi_0)t_\phi t_{\mathrm{ps}}^2\\
&-1536\cos(2\phi-\Delta\mathrm{x}_4-\tan^{-1}(\sqrt{2}))\cos(\chi_0)\sin^3(\chi_0)t_f t_{2\phi}t_{\mathrm{ps}}^2\\
&-256\cos(2\phi-\Delta\mathrm{x}_4-4\chi_0-\tan^{-1}(\sqrt{2}))\cos(\chi_0)\sin^3(\chi_0)t_f t_{2\phi}t_{\mathrm{ps}}^2\\
&-1024\cos(2\phi-\Delta\mathrm{x}_4-2\chi_0-\tan^{-1}(\sqrt{2}))\cos(\chi_0)\sin^3(\chi_0)t_f t_{2\phi}t_{\mathrm{ps}}^2\\
&-256\cos(\chi_0)\cos(2\phi-\Delta\mathrm{x}_4+4\chi_0-\tan^{-1}(\sqrt{2}))\sin^3(\chi_0)t_f t_{2\phi}t_{\mathrm{ps}}^2\\
&-1024\cos(\chi_0)\cos(\Delta\mathrm{x}_4-2(\phi+\chi_0)+\tan^{-1}(\sqrt{2}))\sin^3(\chi_0)t_f t_{2\phi}t_{\mathrm{ps}}^2\\
&+256\cos(\phi-\Delta\mathrm{x}_4-\tan^{-1}(\sqrt{2}))\cos(\chi_0)\sin^2(\chi_0)t_\phi t_{\mathrm{ps}}\\
&+64\cos(\phi-\Delta\mathrm{x}_4-6\chi_0-\tan^{-1}(\sqrt{2}))\cos(\chi_0)\sin^2(\chi_0)t_\phi t_{\mathrm{ps}}\\
&-128\cos(\phi-\Delta\mathrm{x}_4-4\chi_0-\tan^{-1}(\sqrt{2}))\cos(\chi_0)\sin^2(\chi_0)t_\phi t_{\mathrm{ps}}\\
&-64\cos(\phi-\Delta\mathrm{x}_4-2\chi_0-\tan^{-1}(\sqrt{2}))\cos(\chi_0)\sin^2(\chi_0)t_\phi t_{\mathrm{ps}}\\
&-64\cos(\chi_0)\cos(\phi-\Delta\mathrm{x}_4+2\chi_0-\tan^{-1}(\sqrt{2}))\sin^2(\chi_0)t_\phi t_{\mathrm{ps}}\\
&-128\cos(\chi_0)\cos(\phi-\Delta\mathrm{x}_4+4\chi_0-\tan^{-1}(\sqrt{2}))\sin^2(\chi_0)t_\phi t_{\mathrm{ps}}\\
&+64\cos(\chi_0)\cos(\phi-\Delta\mathrm{x}_4+6\chi_0-\tan^{-1}(\sqrt{2}))\sin^2(\chi_0)t_\phi t_{\mathrm{ps}}\\
&-256\cos(\phi)\sin(\chi_0)t_f t_\phi t_{2\phi}t_{\mathrm{ps}}-64\cos(\phi-6\chi_0)\sin(\chi_0)t_f t_\phi t_{2\phi}t_{\mathrm{ps}}\\
&+128\cos(\phi-4\chi_0)\sin(\chi_0)t_f t_\phi t_{2\phi}t_{\mathrm{ps}}+64\cos(\phi-2\chi_0)\sin(\chi_0)t_f t_\phi t_{2\phi}t_{\mathrm{ps}}\\
&+64\cos(\phi+2\chi_0)\sin(\chi_0)t_f t_\phi t_{2\phi}t_{\mathrm{ps}}+128\cos(\phi+4\chi_0)\sin(\chi_0)t_f t_\phi t_{2\phi}t_{\mathrm{ps}}-64\cos(\phi+6\chi_0)\sin(\chi_0)t_f t_\phi t_{2\phi}t_{\mathrm{ps}}\\
&-128\cos(6\chi_0)t_\phi^2+16\cos(8\chi_0)t_\phi^2+\frac{2}{3}((t_{\mathrm{ps}}(21t_{\mathrm{ps}}-10)+21)t_{\mathrm{ps}}^4+840)t_\phi^2\\
&+8\cos(2\chi_0)(t_{\mathrm{ps}}^4+16\sin^2(\chi_0)t_f^2(7t_{\mathrm{ps}}^4+8)t_{2\phi}^2 t_{\mathrm{ps}}^2+t_{\mathrm{ps}}^2+(-t_{\mathrm{ps}}^6+t_{\mathrm{ps}}^4-112)t_\phi^2)+\cos(4\chi_0)((448-17(t_{\mathrm{ps}}^6+t_{\mathrm{ps}}^4))t_\phi^2\\
&-64\sin^2(\chi_0)t_f^2 t_{\mathrm{ps}}^2(t_{\mathrm{ps}}^6-7t_{\mathrm{ps}}^4-4)t_{2\phi}^2-32(t_{\mathrm{ps}}^4+t_{\mathrm{ps}}^2))) \qquad (6)
\end{aligned}
$$

For $\Delta x_4 = 0$ this becomes $\tilde{p}_4 = \min_{x_4}\tilde{p}_4 + 0.64\,(\max_{x_4}\tilde{p}_4 - \min_{x_4}\tilde{p}_4)$ (see Fig. 2(d)).

# Scaling limitations

In this section we discuss the main limitations for the scalability of optical multiport schemes with the architecture similar to that presented in the paper. Below is the list of the main scalability-detrimental factors.

*Restricted phase adjustment precision.*– An $N$-dimensional unitary matrix $U$ can be decomposed into the product of $M = N(N-1)/2$ two-level matrices:

$$U = \prod_{i=1}^{M} T_i, \tag{7}$$

with

$$T_i = \begin{pmatrix} \cos(\phi_i/2) & i\sin(\phi_i/2) \\ i\sin(\phi_i/2) & \cos(\phi_i/2), \end{pmatrix} \tag{8}$$

where $\phi_i$ is the phase determining the split-ratio of the beam splitter. Our setup includes only symmetric beam splitters. However, one can effectively realize an arbitrary split-ratio by utilizing Mach–Zehnder interferometry. This yields the phase $\phi_i$ ($i \in \{1, \ldots, M\}$) adjustment with the precision $\Delta\phi$. We can thus write the total cumulative error in terms of the matrix norms:

$$\|\Delta U\| = \|\sum_{i=1}^{M} T_1 ... T_{i-1}\Delta T_i T_{i+1} ... T_M\| \leq \prod_{i}^{M} \|T_i\| \cdot \left( \sum_{i=1}^{M} \|\frac{\Delta T_i}{T_i}\| \right). \tag{9}$$

The logarithmic derivative

$$\frac{\Delta T_i / T_i}{\Delta \phi_i} = \frac{1}{2} \begin{pmatrix} 0 & i \\ i & 0 \end{pmatrix}. \tag{10}$$

Conversely, a crude estimation gives

$$\frac{\|\Delta U\|}{\prod_{i=1}^{M} \|T_i\|} \leq \sum_{i=1}^{M} \|\frac{\Delta T_i}{T_i}\| \sim \sqrt{M}\Delta\phi \sim N\Delta\phi. \tag{11}$$

In the experiment we had $\Delta\phi \approx 0.2 \div 0.3$. That said, we expect that the use of thinner glass phase shifters with width $d = 0.1\,\mathrm{mm}$ and optical holders with finer control precision $\Delta\alpha \sim 10^{-5}$ ($\alpha$ is the rotation angle of a holder) should enable $\Delta\phi = 10^{-3}$:

$$\Delta\phi = \left( \frac{\partial\phi}{\partial\alpha} \right) \Delta\alpha \approx \frac{2\pi(n-1)d}{\lambda} \frac{\sin\alpha}{\cos\alpha^2} \Delta\alpha \approx 10^{-3}. \tag{12}$$

*Restricted precision of the wavefronts' alignment.*– Let us estimate the error caused by the misalignment of the wavefronts. The signal intensities for the circular- ($I_{\bigcirc}$) and square-shaped ($I_{\square}$) beams are given by

$$I_{\bigcirc} = \iint_S \frac{d^2\mathbf{r}}{\pi R^2} |\sum_{j=1}^{K} \frac{1}{\sqrt{K}} e^{i(\mathbf{k}_j \mathbf{r} - \xi_j)}|^2 = \frac{1}{K} \sum_{i,j=1}^{K} e^{i(\xi_j - \xi_i)} \frac{2J_1(|\mathbf{k}_i - \mathbf{k}_j|R)}{|\mathbf{k}_i - \mathbf{k}_j|R} \sim 1 + I^0_{\bigcirc}(\xi_1, \xi_2, \ldots) \frac{2J_1(\Delta k R)}{\Delta k R}, \tag{13}$$

$$I_{\square} = \iint_S \frac{d^2\mathbf{r}}{a^2} |\sum_{j=1}^{K} \frac{1}{\sqrt{K}} e^{i(\mathbf{k}_j \mathbf{r} - \xi_j)}|^2 = \frac{1}{K} \sum_{i,j=1}^{K} e^{i(\xi_j - \xi_i)} \frac{\sin\left((k_i^{(x)} - k_j^{(x)})a\right)}{(k_i^{(x)} - k_j^{(x)})a} \frac{\sin\left((k_i^{(y)} - k_j^{(y)})a\right)}{(k_i^{(y)} - k_j^{(y)})a}$$
$$\sim 1 + I^0_{\square}(\xi_1, \xi_2, \ldots) \frac{\sin\Delta k a}{\Delta k a}, \tag{14}$$

where the summation is performed over all different trajectories along which the light can travel to the detector through the scheme, the subscript $i$ indicates the number of the trajectory, $K$ is the total number of trajectories incident on the detector which grows exponentially with $N$, $\mathbf{k}_i$ is the wave vector component parallel to the detector's surface, $\xi_i$ is the phase in the center, $\Delta k$ is the characteristic variation of the wave vectors, $R$ and $a$ are, respectively, the radius of the circular-shaped beam and the side length of the square-shaped beam, $J_1$ is the first order Bessel function, $I^0_{\bigcirc}(\xi_1, \xi_2, \ldots)$ and $I^0_{\square}(\xi_1, \xi_2, \ldots)$ are the phase-depended factors. In the ideal case where the wavefronts' misalignment is absent, we have

$$I_{\square(\bigcirc)} \sim 1 + I^0_{\square(\bigcirc)}(\xi_1, \xi_2, \ldots). \tag{15}$$

Thus, the deterioration of the interference picture due to the wavefronts' misalignment is reflected in the factors $\frac{2J_1(\Delta k R)}{\Delta k R}$ and $\frac{\sin\Delta k a}{\Delta k a}$.

Although a scheme realizing an $N$-dimensional unitary matrix comprises of order $N^2$ beam splitters, each trajectory passing only through $\sim N$ of them. Supposing that on passing the $j$th beam splitter the wave vector $\mathbf{k}_i$ diverges for $\mathbf{q}_{i,j}$, and the typical length of $\mathbf{q}_{i,j}$ is $\Delta q$, we get

$$\Delta k \sim \sqrt{N} \Delta q. \tag{16}$$

Here we also assumed that for any $j$ and $m$ such that $j \neq m$, $\mathbf{q}_{i,j}$ and $\mathbf{q}_{i,m}$ are independent. The visibility spoils linearly with the increase of $N$:

$$\frac{2J_1(\Delta kR)}{\Delta kR} \approx 1 - \frac{N(\Delta qR)^2}{8}; \tag{17}$$

$$\frac{\sin \Delta ka}{\Delta ka} \approx 1 - \frac{N(\Delta qa)^2}{6}. \tag{18}$$

If the angle of optical holders is adjusted with the precision $\Delta\alpha$, then $\Delta q = \Delta\alpha \frac{2\pi}{\lambda}$. The equipment employed in our experiment allows for $\Delta\alpha \sim 10^{-5}$ and $R \simeq 1\,\mathrm{mm}$ (or $a = 2\,\mathrm{mm}$); thus, $\frac{2J_1(\Delta kR)}{\Delta kR} \approx 1 - \frac{N\pi^2}{5000}$ and $\frac{\sin \Delta ka}{\Delta ka} \approx 1 - \frac{N\pi^2}{1000}$. One sees that decreasing $R$ or $a$ (which can be easily achieved by the decreasing the detector area), one can further improve the precision.

*Phase fluctuations caused by the surface roughness.–* Assuming that the light acquires delta-correlated random phase $\delta\xi_j(\mathbf{r})$ due to the surface roughness of the optical elements, we can, in a manner similar to the above, write a relation for the signal intensity:

$$I = \langle \iint_S \frac{d^2\mathbf{r}}{\pi R^2} |\sum_{j=1}^{K} \frac{1}{\sqrt{K}} e^{i(\delta\xi_j(\mathbf{r}) - \xi_j)}|^2 \rangle = \frac{1}{K} \sum_{i,j=1}^{K} e^{i(\xi_j - \xi_i)} \iint_S \frac{d^2\mathbf{r}}{\pi R^2} \langle e^{i(\delta\xi_i(\mathbf{r}) - \delta\xi_j(\mathbf{r}))} \rangle \sim 1 + I_0 e^{-N\langle \delta\xi^2 \rangle}, \tag{19}$$

where averaging $\langle \ldots \rangle$ is done over different random phases; we assumed that the beams have a circular shape. We estimate that in our setup $\delta\xi \sim 2\pi/100$; thus, the interference deterioration is given by

$$I \sim 1 + I_0 \exp\left(-\frac{\pi^2 N}{2500}\right). \tag{20}$$

*Intensity losses.–* The intensity losses on the mirrors and beam splitters used in our experiment are about 1%, which is acceptable. By far larger losses ($\approx 10\%$) are associated with the phase shifters. Nevertheless, the use of anti-reflective coating would reduce these losses to 1%. The signal intensity on the detector would be $0.99^N \approx \exp(-N/100)$.

# Fitting problem

## Overview of the problem

In this section, we shall discuss numerical methods which we employ for the analysis of experimental data.

The measurable data consists of four real numbers: the geometrical angle of the swivel platform and three intensities. The fitting curve is determined by Eq. (14) of the main text and depends on the phase vector $\Delta\mathbf{x} = [x_1 - x_1^F, x_2 - x_2^F, x_3 - x_3^F, x_4 - x_4^F]^T$ along with the scaling and shifting parameters $\mathbf{a} = [a_1, a_2, a_3]^T$, $\mathbf{b} = [b_1, b_2, b_3]^T$, $\kappa$ and $\mu$. To fit the experimental data, we apply the method of least squares and search for the minimum of the following loss function:

$$\mathcal{L}(\Delta\mathbf{x}, \mathbf{a}, \mathbf{b}, \kappa, \mu) = \sum_{i=1}^{3} \sum_{j=1}^{N} (p_i(\Delta\mathbf{x}, \kappa \cdot \phi_j + \mu, a_i, b_i) - \mathcal{P}_i(\phi_j))^2, \tag{21}$$

where $\{\mathcal{P}_i(\phi_j)\}_{j=1}^N$ ($i = \{0, 1, 2\}$) is the array of the intensities experimentally measured by $i$th detector with the suffix $j$ representing the number of a data point. As the loss function appears to have multiple local minima, for the purpose of minimization we use the simulated annealing algorithm.

The fitting error is determined by the maximum size of the neighbourhood $\mathcal{O}^F$ of $\mathbf{x}^F$ such that for any $\tilde{\mathbf{x}} \in \mathcal{O}^F$ the standard deviation of $p_i(\tilde{\mathbf{x}}, \phi)$ from $p_i(\mathbf{x}^F, \phi)$ ($i \in \{0, 1, 2\}$) does not exceed the experimental error.

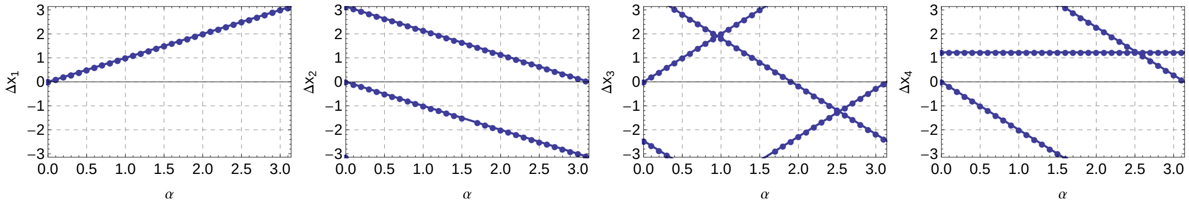


Figure 3: Numerically obtained parameters $\Delta\tilde{\mathbf{x}} = [\Delta\tilde{x}_1, \Delta\tilde{x}_2, \Delta\tilde{x}_3, \Delta\tilde{x}_4]^T$ which for all $\phi$ satisfy the equations $p_i(\Delta\tilde{\mathbf{x}}, \phi) = p_i(\Delta\mathbf{x}_0, \phi)$ as functions of $\alpha$. Here we parameterize $\Delta\mathbf{x}_0(\alpha) = [\alpha, -\alpha, 2\alpha, -2\alpha]^T$. One can see that the same probability distributions $p_i(\phi)$ $(i = \{0, 1, 2\})$ can be realized with two different sets of parameters. Note that by different sets we do not imply those which components differ by $2\pi$.

## Loss landscape

The considered minimization problem has multiple solutions. For instance, Eqs. (11) of the main text and (21) of SI show that the loss function $\mathcal{L}$ is periodical:

$$\mathcal{L}(\Delta\mathbf{x}, \mathbf{a}, \mathbf{b}, \kappa, \mu) = \mathcal{L}(\Delta\mathbf{x} + 2\pi\mathbf{n}, \mathbf{a}, \mathbf{b}, \kappa, \mu) \tag{22}$$

for all integer-valued vectors $\mathbf{n} = [n_1, n_2, n_3, n_4]^T$. In order to find a proper solution of the fitting problem, one also needs to be aware of other patterns concerning the loss function.

The study the landscape of the loss function lies in finding all possible symmetries $S : \mathbb{R}^4 \to \mathbb{R}^4$ such that for all $\Delta\mathbf{x}_0$ and $\phi$ one has

$$p_i(S(\Delta\mathbf{x}_0), \kappa \cdot \phi + \mu, a_i, b_i) = p_i(\Delta\mathbf{x}_0, \kappa \cdot \phi + \mu, a_i, b_i), \tag{23}$$

with $i = \{0, 1, 2\}$. For our investigation, we conduct a series of numerical experiments in which we put $\kappa = 1$, $\mu = 0$, $a_i = 1$, $b_i = 0$ $(i = \{0, 1, 2\})$ (the role of the auxiliary scaling parameters is non-important). In each experiment we parameterize $\Delta\mathbf{x}_0$ using one parameter $\alpha$ and, with $\alpha$ assuming different values, we search for $\Delta\tilde{\mathbf{x}}$ satisfying the relation

$$p_i(\Delta\tilde{\mathbf{x}}, \phi) \equiv p_i(\Delta\mathbf{x}_0, \phi). \tag{24}$$

We then plot elements of $\Delta\tilde{\mathbf{x}}$ as functions of $\alpha$ and identify possible symmetries corresponding to Eq. (23). For each experiment the parameterization is chosen differently, which ensures that none of the symmetries are missed out.

Based on the obtained numerical data, we may suggest that aside from the trivial case of $2\pi$-periodicity there also exist only one linear symmetry given by

$$S\begin{bmatrix} x_1 \\ x_2 \\ x_3 \\ x_4 \end{bmatrix} = \begin{bmatrix} x_1 \\ x_2 + \pi \\ -x_3 + 4\arctan(\sqrt{2}) \\ x_3 + x_4 + \pi - 2\arctan(\sqrt{2}) \end{bmatrix}. \tag{25}$$

Note that $S(S(\Delta\mathbf{x})) = \Delta\mathbf{x} + [0, 2\pi, 0, 2\pi]^T$. The symmetry can be particularly seen in Fig. 3 in which we put $\Delta\mathbf{x}_0(\alpha) = [\alpha, -\alpha, 2\alpha, -2\alpha]^T$. The vertical axis corresponds to the elements of $\Delta\tilde{\mathbf{x}}$ satisfying Eq. (24), whereas the abscissa is the parameter $\alpha$. The manifestation of the symmetry $S$ can be observed in the lines additional to those which correspond to $\Delta\mathbf{x}_0(\alpha)$.

Assuming that the alignment process is relatively precise, the knowledge of symmetry gives us additional leverage for solving the fitting problem. After numerically finding the global minimum of $\mathcal{L}$ with the corresponding parameter vector $\Delta\mathbf{x}$, we can check whether there exists another one with the parameters $\Delta\mathbf{x}'$ such that $|\Delta\mathbf{x}'| < |\Delta\mathbf{x}|$. This can be done by comparing $\Delta\mathbf{x}$ with $\Delta\mathbf{x}' = S(\Delta\mathbf{x}) + 2\pi\mathbf{n}$ for various integer-valued vectors $\mathbf{n}$. As a result, we obtain fitting parameters which are, considerably, the most consistent with the intended Fourier transform.
